# Engineered lipid nanoparticles with synergistic dendritic cell targeting and enhanced endosomal escape for boosted mRNA cancer vaccines

**DOI:** 10.1016/j.mtbio.2025.102107

**Published:** 2025-07-19

**Authors:** Sizhen Wang, Jianyu Zheng, Jiao Zhou, Weiwei Jiang, Zhendong Chen, Xiaoxian Wu, Beibei Guo, Yanfeng Wu, Feng Yang

**Affiliations:** a National Key Laboratory of Immunity and Inflammation & School of Pharmacy, Naval Medical University, Shanghai, 200433, China; b National Key Laboratory of Immunity and Inflammation & Institute of Immunology, College of Basic Medical Sciences, Naval Medical University, Shanghai, 200433, China

**Keywords:** Lipid nanoparticle, mRNA therapy, Antitumor immunology, DC-Targeting ability, Endosomal escape

## Abstract

Lipid nanoparticles (LNPs) have emerged as a pivotal carriers for enhancing mRNA therapeutics, particularly in antitumor therapy. However, achieving robust antigen expression remains a major challenge due to limitations in precise targeted delivery and inefficient endosomal escape. In this study, we constructed an ambidextrous LNP to achieve robust tumor-specific antigen expression through targeted cell delivery and enhanced endosomal escape of mRNA-LNPs. To improve endosomal escape, we synthesized a novel pH-responsive PEGylated lipid designed to synergistically enhance membrane fusion effect with ionizable lipids, thereby optimizing the translation of the desired antigen. This is accomplished by promoting the early endosomal escape and endosomal recycling transport. For precise delivery of mRNA-LNPs to dendritic cells, we employed a mannose-modified PEGylated lipid that targets mannose receptors. Our results demonstrated that the combination of mannose-directed targeting and pH-mediated endosomal escape significantly enhances antigen translation and expression, leading to a vigorous immune response, as validated by both in vitro and in vivo experiments. This ambidextrous strategy advances the formulation of LNPs for precise mRNA delivery and effective antigen encoding, facilitating the development of mRNA vaccines in the field of antitumor immunology.

## Graphical abstract

**Figure:**
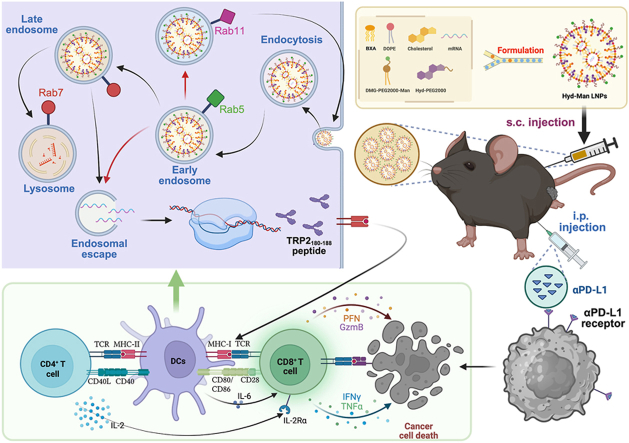

## 1 Introduction

The emergence of COVID-19 mRNA vaccines has catapulted mRNA therapy into the forefront of biomedical research, particularly within the domain of tumor immunotherapy [[1], [2], [3]]. Leveraging advanced mRNA technology, it is now possible to deliver tailored mRNA sequences into immune cells to express specific tumor antigens. This process can stimulate autologous tumor-specific immune responses and enhance tumor treatment when combined with immune checkpoint inhibitors, heralding a new era for addressing refractory tumors [[4], [5], [6]].

mRNA therapy offers several advantages, including the rapid design and production of target proteins, scalability for mass manufacturing, and transient expression [[7], [8], [9]]. However, persistent challenges related to mRNA stability, delivery efficiency, cellular targetability, and antigen expression must be addressed to unlock the full potential of mRNA therapeutics [[10], [11], [12]]. To realize this potential in tumor immunotherapy, it is essential to modulate mRNA molecules and develop efficient delivery systems [13,14]. Recently, lipid nanoparticles (LNPs) have emerged as a promising nonviral platform for mRNA delivery, as evidenced by the successful applications in the Moderna and Pfizer-BioNTech vaccines against COVID-19 [[15], [16], [17], [18]].

LNPs harness similarities in hydrophobicity with cellular membranes, enhancing their drug delivery capabilities. Additionally, mRNA encapsulated in LNPs exhibits greater stability compared to naked mRNA, minimizing degradation by nucleases [19]. Significant advancements, such as nucleotide modifications pioneered by Karikó and Weissman, have further improved the stability, immunogenicity, and translation efficacy of mRNA [[20], [21], [22]]. Despite these remarkable developments, a critical challenge that remains is insufficient protein encoding resulting from both the precise delivery to desired cells and the limited release of nucleic acid payloads in the cytoplasm [[23], [24], [25], [26]]. This shortfall contributes to inadequate tumor-specific immune responses.

Dendritic cells (DCs), as key antigen-presenting cells (APCs), play a pivotal role in activating naïve T cells by cross-presenting antigens via major histocompatibility complex class I (MHC-I) or class II (MHC-II) molecules [[27], [28], [29], [30]]. A single DC can activate 100–200 T cells, highlighting their importance in orchestrating immune responses [31]. Given their central role, targeting DCs for vaccine development presents a promising strategy for tumor immunotherapy, as they can significantly inhibit tumor growth and induce long-term protective immunity through memory T cell activation [[32], [33], [34]]. The abundant expression of mannose receptors on DCs makes mannose a well-established target for enhancing LNP-mediated delivery of mRNA, thereby improving antigen presentation and subsequent immune responses.

While targeting DCs is a vital strategy, it alone is insufficient to optimize delivery efficiency and ensure adequate protein encoding. Studies have demonstrated that a novel type of N^1^,N^3^,N^5^-tris(2-aminoethyl)benzene-1,3,5-tricarboxamide-derived LNPs exhibits exceptional transfection efficiency and is capable of effectively delivering various types of mRNA [35,36]. Additionally, effective endosomal escape of mRNA-LNPs is crucial, as the cytoplasm is the primary site for mRNA translation [[37], [38], [39], [40]]. Upon internalization, LNPs traverse early and late endosomes before reaching lysosomes, where enzymatic degradation is prevalent [41,42]. To achieve successful cellular outcomes, mRNA must be released into the cytosol prior to lysosomal degradation, a process known as endosomal escape [26,43]. However, this step remains inefficient and is a significant bottleneck in LNP-based delivery systems [[44], [45], [46]]. Previous studies indicate that a considerable proportion of internalized LNPs either undergo lysosomal degradation or extracellular recycling, limiting the release of nucleic acid payloads [47,48].

To overcome these challenges and achieve precise delivery and robust protein expression for a potent immune response, a dual approach that incorporates both targeted design and enhanced endosomal escape is paramount. However, the integration of efficient DC targeting with improved endosomal escape using a single nanocarrier presents a formidable challenge [31,49].

In this study, we propose an ambidextrous strategy with engineered lipid nanoparticle to enable potent tumor-specific antigen expression through targeted delivery and enhanced endosomal escape of mRNA. We synthesized an imitative N^1^,N^3^,N^5^-tris(2-aminoethyl)benzene-1,3,5-tricarboxamide-derived LNP (BXA LNP) system tailored for efficient mRNA delivery. Our approach features a unique pH-responsive PEGylated lipid with a hydrazone bond designed to undergo cleavage under weakly acidic conditions, thereby exposing amino groups. This exposure facilitates protonation and promotes the fusion of LNPs with endosomal membranes, optimizing mRNA release. Moreover, the pH-responsive lipid reduces steric hindrance in the acidic environment of endosomes, enhancing the translocation of mRNA to the cytosol.

For targeted delivery to DCs, we incorporated mannose-modified PEGylated lipids as ligands for mannose receptors. We then replaced traditional PEGylated lipids with our innovative pH-responsive variants to formulate a dual-action mRNA-LNP system capable of precise delivery and effective endosomal escape (Hyd-Man LNPs). This innovative ambidextrous approach significantly boosts the translational and encoding efficacy of nucleic acid molecules, paving the way for the design of mRNA cancer vaccines that elicit strong immune responses, exemplified by the use of the TRP2_180–188_ peptide (SVYDFFVWL) as a representative antigen. Additionally, when combined with an immune checkpoint inhibitor (anti–PD-L1 monoclonal antibody), our system exhibited a synergistic effect, further augmenting antitumor activity.

## 2 Materials and methods

### 2.1 Materials

The ionizable lipid DLin-MC3-DMA, 1,2-dioctadecanoyl-sn-glycero-3-phosphocholine (DSPC), and cholesterol (Chol) were purchased from AVT Pharmaceutical Tech Co., Ltd (Shanghai). 1,2-Dioleoyl-sn-glycero-3-phosphoethanolamine (DOPE) and Cy5-Cholesterol were sourced from Xi'an Ruixi Biological Technology Co., Ltd (Xi'an). Benzaldehyde-mPEG_2000_ (mPEG_2000_-DF, R-BJQPE-2k) was pyrchased from Xi'an ruixi Biological Technology Co., Ltd, China. The mCherry mRNA, Cy5-mCherry mRNA and TRP2_180-188_ mRNA were obtained from Humantech Co., Ltd (Shanghai). The ionizable lipid BXA and pH-responsive hydrazone-based mPEG_2000_ lipid (Hyd-mPEG_2000_) were synthesized in our laboratory (details available in Supplementary Information). Rab5A #2143, Rab7 #9367, and Rab11 #5589 Rabbit mAb were purchased from Cell Signaling Technology (USA). Alexa Fluor®594-conjugated AffiniPure Goat Anti-Rabbit IgG (H + L) #152106 was purchased from Jackon ImmunoResearch (USA). TRP2 primary antibodies #04101 was sourced from GeneTex (USA) and anti-rabbit IgG H&L (HRP) #6721 was from abcam (USA). TRP2_180-188_ peptide (SVYDFFVWL) was purchased from Sangon Biotech Co., Ltd (Shanghai).

### 2.2 Preparation of mRNA-Encapsulated LNPs

mRNA-encapsulated LNPs were prepared using microfluidic mixing [50]. A lipid mixture was dissolved in ethanol at a specific molar ratio, while mRNA was dissolved in RNase-free citrate buffer (100 mM, pH 4.0). The aqueous mRNA solution was rapidly mixed with the lipid mixture at a 3:1 volumetric ratio (total flow rate of 15 mL/min) using a microfluidic mixing device (INano TME, Micro&Nano, Shanghai, China), maintaining a nitrogen-to-phosphorus ratio of 3 (N/P = 3). The molar ratios for Hyd-Man LNPs were as follows: BXA/Chol/DOPE/DMG-mPEG_2000_-Man/Hyd-mPEG_2000_ at 30/40/30/0.375/1.125. The lipid compositions of BXA LNPs and BXA-Man LNPs were BXA/Chol/DOPE/DMG-mPEG_2000_ (30/40/30/0.375) and BXA/Chol/DOPE/DMG-mPEG_2000_-Man (30/40/30/0.375), respectively. The commercial MC3 LNP was formulated as DLin-MC3-DMA/Chol/DSPC/DMG-mPEG_2000_ (50/38.5/10/1.5). For fluorescent labeling, Cy5-mCherry mRNA was used to prepared LNP in the tests in vitro, while 1.5 % molar of cholesterol was substituted with equimolar Cy5-cholesterol in the cellular uptake test in vivo. After preparation, LNPs were immediately diluted with 10 times the volume of 1 × PBS and concentrated using Millipore Amicon Ultra-15 (100 kDa, Milipore). The final mRNA-encapsulated LNP solution was stored at 4 °C for subsequent experiments.

### 2.3 Characterization

Particle size and zeta potential of LNPs were measured using a Malvern Zetasizer Nano ZSE. The morphology of LNPs was observed using transmission electron microscopy (TEM, Tecnai G2 F20 S-Twin). Encapsulation efficiency was assessed with the Quanti-it™ RiboGreen RNA assay kit (Thermo Fisher Co., Ltd). RNA concentrations in samples treated with Triton X-100 (to measure total RNA content) and untreated samples (to quantify unencapsulated mRNA) were recorded. Encapsulation efficiency was calculated using the formula:

Encapsulation Efficiency (EE%) = (C_T_-C_U_)/C_T_ × 100 %

where C_T_ is the total RNA concentration and C_U_ is the concentration of unencapsulated mRNA.

### 2.4 Measurement of hydrolysis rate of Hyd-mPEG_2000_ in different pH environments

Hydrolysis rates were assessed by detecting primary amine groups with TNBS [51]. Hyd-mPEG_2000_ (0.1 g) was diluted in buffers of varying pH (pH 5.5, 6.5, 7.4 in 0.1 M PBS) and incubated at 37 °C with shaking (200 rpm). Following incubation, samples were treated with TNBS to measure absorbance at 420 nm. The hydrolysis rate was calculated using:

Hydrolysis Rate (HR%) = (A_H_-A_H0_) / (A_T_-A_T0_) × 100 %

A_H_ is the absorbance of Hyd-mPEG_2000_ hydrolyzed over time, A_H0_ the absorbance of the blank solvent, A_T_ the absorbance of Compound 4 (Supplementary Information), and A_T0_ the blank absorbance.

### 2.5 Cellular uptake In vitro

DC2.4 cells were seeded (1 × 10^5^/well) in complete medium for 12 h. Various Cy5-labeled mCherry-encapsulated LNPs (600 ng/mL mRNA) were co-incubated with cells for 1 h and 4 h. Cells were subsequently stained with Hoechst 33342 for 10 min, washed three times with PBS, and analyzed using confocal microscopy (CLSM, Zeiss LSM 710).

### 2.6 Cell viability In vitro

To assess cytotoxicity of Hyd-Man mRNA-encapsulated LNPs, DC2.4 cells (1 × 10^4^/well) and BMDCs (1 × 10^4^/well in U-bottom plates) were cultured for 12 h, then incubated with mRNA-encapsulated LNPs at various concentrations for 24 h. Cell viability was assessed using the CCK-8 kit.

### 2.7 DC-targeting performance and transfection assays In vitro

DC2.4 cells (1 × 10^5^/well), BMDCs (5 × 10^5^/well) and 293T cells (1 × 10^5^/well) were treated with different mRNA-encapsulated LNPs (600 ng/mL) for 24 h mCherry protein absorbance was quantified using a multifunction reader (SpectraMax3, Molecular Devices) (Ex/Em = 590/645), and TRP2_180-188_ transfection was measured by dot blot using specific primary and secondary antibodies.

### 2.8 Endosomal escape assessment via colocalization with acidic organelles

DC2.4 cells (1 × 10^5^/well) were seeded and treated with Cy5-labeled mCherry mRNA-encapsulated LNPs (600 ng mRNA/mL) for 1 h or 4 h. Cells were stained with LysoTracker™ Green DND-26 (50 nM) and Hoechst 33342 before imaging via CLSM. JACoP and Plot Profile tools were utilized for quantifying LNP and organelle association.

### 2.9 Endosomal escape assessment via calcein release

The calcein release assay was performed as previously described [52]. DC2.4 cells were seeded, washed with PBS, and treated with fresh medium containing 100 μM calcein and various mRNA-encapsulated LNPs (600 ng/mL) for 2 h. Cells were further stained and examined via CLSM.

### 2.10 Endosomal trafficking assay by immunofluorescence

For endosomal protein analysis, DC2.4 cells (2 × 10^5^/well) were treated with TRP2_180-188_ mRNA-encapsulated LNPs (600 ng mRNA/mL) for 4 h. Cells were fixed, permeabilized, and incubated with primary antibodies against endosomal markers overnight. After washing, cells were incubated with a secondary antibody and observed through CLSM.

### 2.11 Endosomal trafficking assay by western blot

DC2.4 cells (1 × 10^6^/well) were treated with TRP2_180-188_ mRNA-encapsulated LNPs (600 ng mRNA/mL) for 4 h. Total protein was extracted, separated by SDS-PAGE, and transferred to PVDF membranes. Following blocking, membranes were incubated with primary antibodies overnight, washed, and treated with secondary antibodies for visualization via ECL.

### 2.12 Immune activation In vitro

BMDCs transfected with TRP2_180-188_ mRNA-encapsulated LNPs (600 ng mRNA/mL) for 24 h were co-cultured with SLCs at a 1:5 ratio for an additional 24 h. Supernatants were collected and cytokine levels (TNF-α, IFN-γ, Granzyme B) were quantified using ELISA kits. T cell populations were identified via flow cytometry.

### 2.13 Specific cell-killing test In vitro

Cytotoxic T lymphocyte (CTL) activity was evaluated using the CCK-8 and LDH assays. B16-F10 cells were pre-seeded in 96-well plates. BMDCs were incubated with TRP2_180-188_ mRNA-encapsulated LNPs for 24 h, and then co-cultured with SLCs and B16-F10 cells (1:5:1) for an additional 24 h or 48 h. Supernatants were analyzed for LDH release, and remaining attached B16-F10 cells were assessed for viability using the CCK-8 kit.

### 2.14 Treatment of B16-F10 tumors In vivo

In this study, all animals were treated in accordance with the Guiding Principles for the Committee on Ethics of Medicine, Naval Medical University.

Male C57BL/6 mice (20–22 g) were subcutaneously implanted with 1 × 10^5^ B16-F10 cells. Upon reaching 80–100 mm^3^, mice were treated with PBS or 5 μg of TRP2_180-188_ mRNA encapsulated in LNPs on days 0 and 7. Combinatorial immunotherapy groups received intraperitoneal injections of 100 μg anti-PD-L1 mAb. Tumor sizes were measured, and tissues collected for H&E and immunofluorescence staining after euthanasia on day 14.

### 2.15 Flow cytometry analysis

Flow cytometric analysis utilized antibodies to CD45, CD3, CD4, CD8a, CD11c, CD80, and CD86 obtained from Thermo Fisher Scientific. Tumor tissues were processed, single-cell suspensions prepared, and analyzed for immune cell counts and surface marker expression using a SONY flow cytometer.

### 2.16 ELISA assay

Tumor tissues (0.2 g) were homogenized in ice-cold lysis buffer, centrifuged, and total protein concentration determined via BCA kit. Cytokine levels (GzmB, TNF-α, IFN-γ) in tumor tissues were quantified using ELISA kits, normalizing by weight and total protein concentration.

### 2.17 Statistical analysis

Statistical analyses were performed using GraphPad Prism 10.0. Data are presented as mean ± SD. Statistical significance between groups was analyzed using one-way ANOVA, with significance defined at ∗p < 0.05, ∗∗p < 0.01, ∗∗∗p < 0.001, and ∗∗∗∗p < 0.0001. The number of animals used is specified in each figure.

## 3 Results and discussion

### 3.1 Synthesis of Hyd-Man LNP for mRNA delivery

**Fig. 1:**
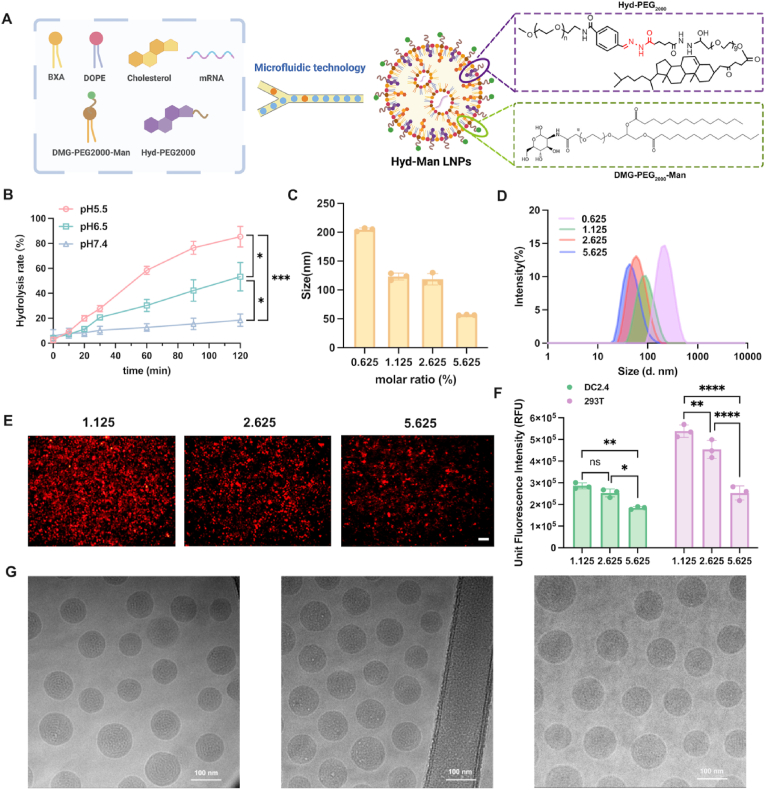
Synthesis and characterization of Hyd-Man LNPs. (A) Flowchart of Hyd-Man LNPs synthesis by microfluidic devices. Created with BioRender.com. (B) Hydrolysis rate of Hyd-mPEG_2000_ in different pH environments (n = 3). (C) Particle size of Hyd-Man LNPs with different molar ratio of Hyd-mPEG_2000_ (Hyd-Man-0.625/1.125/2.625/5.625). (D) The size distribution of Hyd-Man-0.625/1.125/2.625/5.625 LNPs detected by DLS. (E) Transfection efficiency of mCherry mRNA-encapsulated Hyd-Man LNPs with different molar ratio of Hyd-mPEG_2000_ in DC2.4 and 293T cells (n = 3). (F) Transfection of mCherry mRNA-encapsulated Hyd-Man LNPs with different molar ratio of Hyd-mPEG_2000_ in 293T cells detected by fluorescence microscope. Scale bar = 50 μm. (G) Cryo-TEM images of Hyd-Man LNPs. Scale bar = 100 nm. Significant differences were assessed using a one-way ANOVA with Tukey test (∗p < 0.05; ∗∗p < 0.01; ∗∗∗p < 0.001; ∗∗∗∗p < 0.0001). Data were presented as mean ± SD.

The synthesis of the BXA ionizable lipid was conducted according to established protocols [48]. We successfully synthesized both the BXA ionizable lipid and Hyd-mPEG_2000_ PEGylated lipid, following the representative synthetic pathways depicted in Fig. S1 and S2. Their chemical structures were characterized using mass spectrometry (MS) and nuclear magnetic resonance (NMR) spectroscopy (Fig. S3–S5). Importantly, the original pH-responsive Hyd-mPEG_2000_ was a novel PEGylated lipid formed by connecting two PEG chains via a linker bond (Fig. 1A). One chain was DMG-PEG_400_-amine (Compound 4, short-chain PEG), while the other was benzaldehyde-mPEG_2000_ (long-chain PEG). These two chains were combined through an acylhydrazone bond, which exhibited pH-responsive characteristics [53,54].

The initial formulation of BXA LNPs was achieved by combining BXA ionizable lipid with cholesterol, DOPE, and DMG-mPEG_2000_ via microfluidic mixing. Subsequently, we replaced DMG-mPEG_2000_ with DMG-mPEG_2000_-Man to create mannose-modified LNPs (Man LNPs). To synthesize Hyd-Man LNPs, we incorporated a portion of Hyd-mPEG_2000_ into the Man LNPs, resulting in reduced particle size and enhancing targeted mRNA delivery to DCs (Fig. 1A).

To investigate the pH-responsiveness of Hyd-mPEG_2000_, we assessed its hydrolysis rates across various pH environments. As illustrated in Fig. 1B, a significant difference in hydrolysis rates was observed after 2 h of incubation, with the rate at pH 5.5 notably higher compared to pH 6.5 and pH 7.4 (85.41 ± 8.27 % vs. 53.28 ± 11.33 % vs. 18.47 ± 4.96 %). This finding confirms the pH-sensitive behavior of Hyd-mPEG_2000_.

A systematic approach was employed to evaluate the optimal ratio of Hyd-mPEG_2000_ by preparing Hyd-Man LNPs with varying percentages: 0.625 %, 1.125 %, 2.625 %, and 5.625 %. LNPs incorporating 0.625 % Hyd-mPEG_2000_ exhibited poor particle size greater than 200 nm, leading to its exclusion from subsequent experimental evaluations (Fig. 1C and D). The transfection efficiencies of LNPs modified with different molar ratios of Hyd-mPEG_2000_ (1.125 %, 2.625 %, 5.625 %) are presented in Fig. S6A. Notably, LNPs containing 1.125 % Hyd-mPEG_2000_ (designated as Hyd-Man LNPs) demonstrated the most effective transfection capabilities in DC and 293T cells (Fig. 1E and F), indicating their suitability for mRNA vaccine applications.

The final Hyd-Man LNP formulation exhibited spherical nanostructures with an average diameter of approximately 120 nm (Fig. 1G–Table S1). Importantly, these LNPs demonstrated a high encapsulation efficiency (EE%) exceeding 85 %, as indicated in Table S1. The physicochemical properties of other LNPs used for comparative studies are also summarized in Table S1.

### 3.2 Hyd-Man LNPs demonstrate enhanced cellular uptake and transfection efficiency

**Fig. 2:**
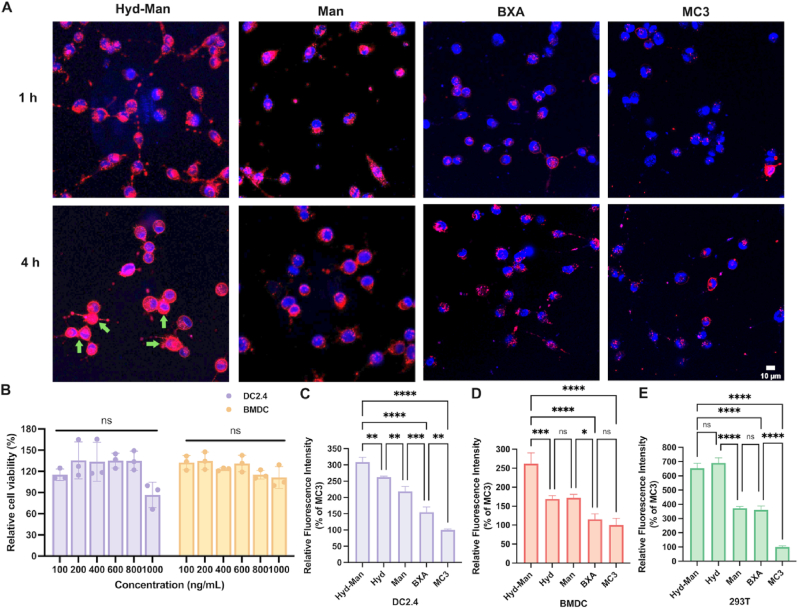
Cellular uptake and transfection efficiency. (A) Cellular uptake of Cy5-labeled mCherry mRNA encapsulated in different LNPs (red) was evaluated at 1 h and 4 h. Scale bar = 10 μm. (B) Cytotoxicity of Hyd-Man LNPs was evaluated in DC2.4 cells (n = 3). Transfection efficiency of LNPs encapsulated mCherry mRNA in (C) DC2.4 cells, (D) BMDCs and (E) 293T cells (n = 3). Significant differences were assessed using a one-way ANOVA with Tukey test (∗p < 0.05; ∗∗p < 0.01; ∗∗∗p < 0.001; ∗∗∗∗p < 0.0001). Data were presented as mean ± SD. (For interpretation of the references to colour in this figure legend, the reader is referred to the Web version of this article.)

We evaluated the cellular uptake of different LNPs loaded with Cy5-mCherry mRNA using confocal laser microscopy. Notably, Hyd-Man LNPs exhibited superior cellular uptake compared to both BXA and MC3 (commercial LNP) formulations within just 1 h (Fig. 2A), confirming the targeting capability of mannose for DCs. Remarkably, the Hyd-Man LNPs group displayed exceptional uptake across all conditions, attributed to the mannose modification facilitating DC targeting and the Hyd-mPEG_2000_ component promoting mRNA endosomal escape. This enhanced release of mRNA into the cytoplasm led to the expedited expression of mCherry proteins within 4 h, resulting in widespread red fluorescence throughout the cells (indicated by green arrows).

Prior to in vitro transfection, we assessed the cytotoxicity of mCherry mRNA Hyd-Man LNPs in DC2.4 and bone marrow-derived dendritic cells (BMDCs) by measuring cell viability following treatment with various LNP doses over 24 h. The Hyd-Man LNPs demonstrated excellent biocompatibility, with cell viability remaining comparable even at mRNA concentrations up to 1000 ng/mL (Fig. 2B).

Subsequent analysis revealed that Hyd-Man LNPs achieved the outstanding transfection efficiency in both DC cells (DC2.4 and BMDCs) and 293T cells (Fig. 2C–E). The relative fluorescence intensity of mCherry proteins transfected by Hyd-Man LNPs was significantly greater than those of both Hyd LNPs and Man LNPs in DC cells, underscoring the potential synergistic effect of the mannose-directed DC targeting and pH-enhanced endosomal escape strategy. Importantly, Man LNPs exhibited 1–2 times the fluorescence intensity of BXA LNPs in DC cells, while showed no significant difference in 293T cells, reaffirming the advantages of mannose for DC-targeted mRNA vaccines. Furthermore, compared with BXA LNPs, Hyd LNPs demonstrated significantly higher transfection efficiency, verifying the feasibility of the pH-enhanced mRNA endosomal escape strategy. The synergetic effect of pH-mediated enhanced endosomal escape and mannose-directed targeting led to a fluorescence intensity in the Hyd-Man LNPs group that was approximately double that of BXA and three times that of MC3 LNPs in DC2.4 cells, and two-fold higher in BMDCs, respectively. Dot blot analyses further corroborated the effective transfection of Hyd-Man LNPs encapsulated TRP2_180-188_ mRNA, aligning with the mCherry transfection results (Fig. S6B and S6C).

### 3.3 Efficient endosomal escape of Hyd-Man LNPs

**Fig. 3:**
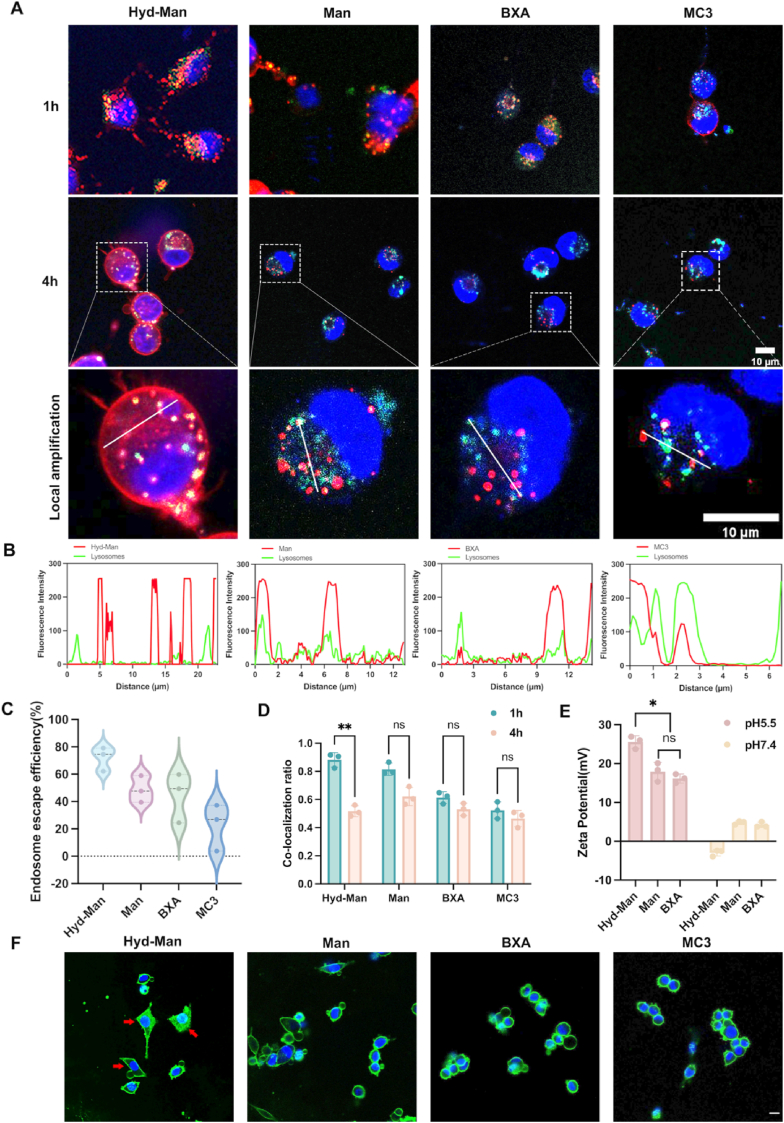
Endosomal escape of Hyd-Man LNPs. (A) Colocalization of Cy5-labeled mCherry mRNA-encapsulated LNPs (red) with lysosome (green) at 1 h and 4 h. Scale bar = 10 μm. (B) Plot profile analysis of local amplification at 4 h. (C) Endosomal escape efficiency of mRNA in different LNPs calculated by Plot profile analysis in local amplification (n = 3). (D) Percentage of colocalization between mRNA-LNPs and lysosomes at 1 h and 4 h through JACoP by Image J software (n = 3). (E) Zeta potential of different LNPs incubated in different pH environment (pH 5.5 and 7.4). (F) Cytosolic localization of calcein (green) in DC2.4 cells. Nuclei were stained using Hoechst 33342 (blue). Scale bar = 10 μm. Significant differences were assessed using a one-way ANOVA with Tukey test (∗p < 0.05; ∗∗p < 0.01; ∗∗∗p < 0.001; ∗∗∗∗p < 0.0001). Data were presented as mean ± SD. (For interpretation of the references to colour in this figure legend, the reader is referred to the Web version of this article.)

Following our confirmation of cellular uptake, we investigated endosomal escape via subcellular distribution using CLSM imaging after 1- and 4-h treatments with Cy5-labeled mRNA encapsulated in different LNPs (Fig. 3A). The local amplification analyzed by Plot Profile was exhibited the optimal endosomal escape ability of Hyd-Man LNP among these LNP groups, with endosomal escape efficiency over 60 %. In contrast, other formulations showed higher colocalization rates ranging from 20 % to 50 % (Fig. 3B and C). Besides, the Hyd-Man LNP demonstrated significantly decreased colocalization with LysoTracker Green-stained acidic organelles, indicating successful pH-mediated release of mRNA from endosomes between 1 and 4 h, while other groups displayed minimal release within this time frame (Fig. 3D–S7).

To further assess endosomal escape, we co-incubated mRNA-encapsulated LNPs with calcein for 2 h. Due to calcein's membrane-impermeable nature, its diffusion into the cytosol serves as a marker for endosomal membrane disruption [52]. As illustrated in Fig. 3F, the Hyd-Man LNPs facilitated significant calcein diffusion into the cytosol of DC2.4 cells, confirming effective endosomal escape. Additionally, ζ-potential measurements indicated that Hyd-Man LNPs exhibited a favorable positive charge in acidic environments (Fig. 3E), enhancing electrostatic interactions that promote endosomal escape. Because in the acidic microenvironment of endosomes, the Hyd-mPEG_2000_ component dissociates from the LNPs after the acylhydrazone bond cracking, fully exposing the amine group (-NH_3_), which becomes protonated, leading to enhance ζ-potential.

### 3.4 Endosomal trafficking of Hyd-Man LNPs

**Fig. 4:**
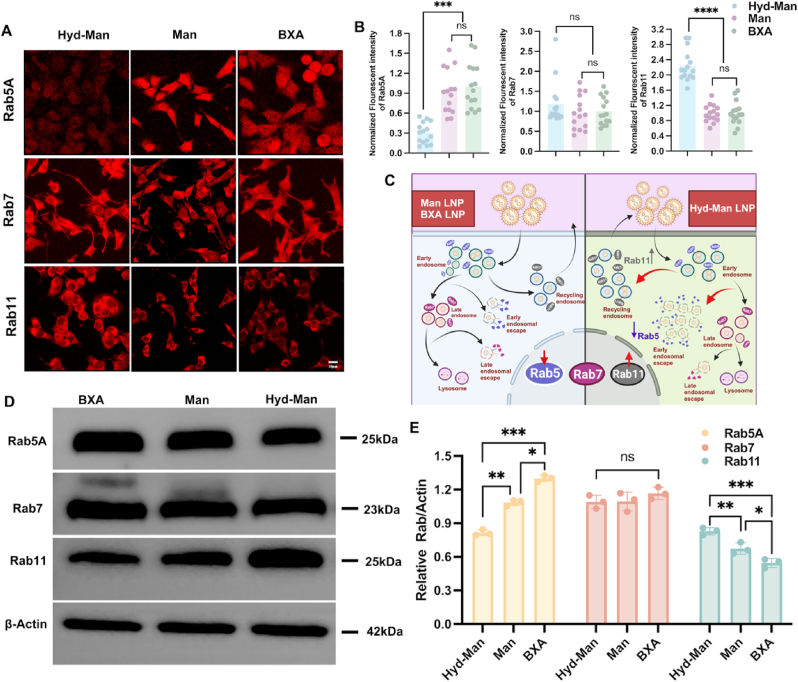
Characterization of LNPs endosomal trafficking. (A) Confocal microscopy images of DC2.4 stained with antibodies for Rab5, Rab7, and Rab11. Cells were treated with Hyd-Man, Man, and BXA LNPs at 600 ng mRNA/mL. (B) Rab5, Rab7, and Rab11 expression was quantified by averaging the fluorescent signal from at least 15 cells in each treatment group. (C) Schematic diagram of the LNPs endosomal trafficking through the expression of Rab protein. Created with BioRender.com. (D) Western blot analysis of Rab5, Rab7, and Rab11 expression in DC2.4 after treated with different TPR2_180-188_ mRNA-encapsulated LNPs for 4 h. β-actin was set as loading control. (E) Quantitation of relative protein concentration in DC2.4 treated with indicated mRNA LNPs. Significant differences were assessed using a one-way ANOVA with Tukey test (∗p < 0.05; ∗∗p < 0.01; ∗∗∗p < 0.001; ∗∗∗∗p < 0.0001). Data were presented as mean ± SD.

To elucidate the effects of Hyd-Man LNPs on endosomal trafficking, we analyzed the involvement of Rab proteins (Rab5, Rab7, and Rab11). Rab5, associated with early endosomes, regulates LNPs into vesicular and transport, while Rab7 is linked to late endosomes, mediating lysosomal interactions. Rab11, involved in recycling endosomes, plays a crucial role in the recycling and reuse of internalized LNPs. After treating DC2.4 cells with Hyd-Man, Man, and BXA LNPs for 4 h, confocal imaging revealed distinctive trends in Rab protein expression (Fig. 4A and B). Specifically, Rab5 expression was reduced, while Rab11 levels increased in the Hyd-Man LNPs group compared to the other formulations. In view of this phenomenon, we put forward an assumption (Fig. 4C): In the Hyd-Man LNPs group, the introduction of pH-responsive performance further activated the early endosomal escape of mRNA, thus promoting membrane fusion between LNP and early endosome, resulting in destruction of endosomal membrane and down-regulation of Rab5 protein expression. While up-regulate Rab11 protein expression, promoting the recycling pathway, achieving the recovery and reuse of mRNA-LNP and preventing its degradation through the lysosomal pathway, which increased the chance of mRNA release. Additionally, there was no significant difference in the expression of Rab7 protein-labeled late endosomes among all LNP groups, suggesting that the intensity of mRNA-LNP entering the late endosome transport was comparable in all groups.

Given Rab11's well-established dual role in endosomal recycling and exocytosis, we proposed the existence of a dynamic intracellular balance between LNP recycling and excretion. When this balance shifted toward enhanced recycling—either due to pH-sensitive cleavage of PEG-lipids or improved interaction with early endosomal regulators—LNPs were retained longer in endosomal compartments conducive to escape. Our data supported this hypothesis: when the recycling pathway was predominant, we observed significantly increased mCherry mRNA expression, more efficient endosomal escape (as seen in cellular uptake and subcellular co-localization studies), and higher levels of calcein release.

Therefore, the DC-targeted/pH-responsive Hyd-Man LNP may activate the early endosome escape pathway, promoting the fusion and rupture of the early endosome membrane, and enabling the release of mRNA from the early endosome into the cytoplasm for expression, while it enhances the endosome recycling transport, facilitating the recovery and reuse of LNP, wherein pH-responsiveness plays a critical regulatory role. This assumption was also supported by Western blot analysis, which demonstrated that Rab5/Rab11 expression after pH-responsive modulation (∗∗p < 0.01, Hyd-Man *vs.* Man) was significantly greater than that achieved through mannose modification-mediated modulation (∗p < 0.05, Man *vs.* BXA) (Fig. 4D, E and S8).

### 3.5 Immunostimulatory effects of Hyd-Man LNPs in vitro

**Fig. 5:**
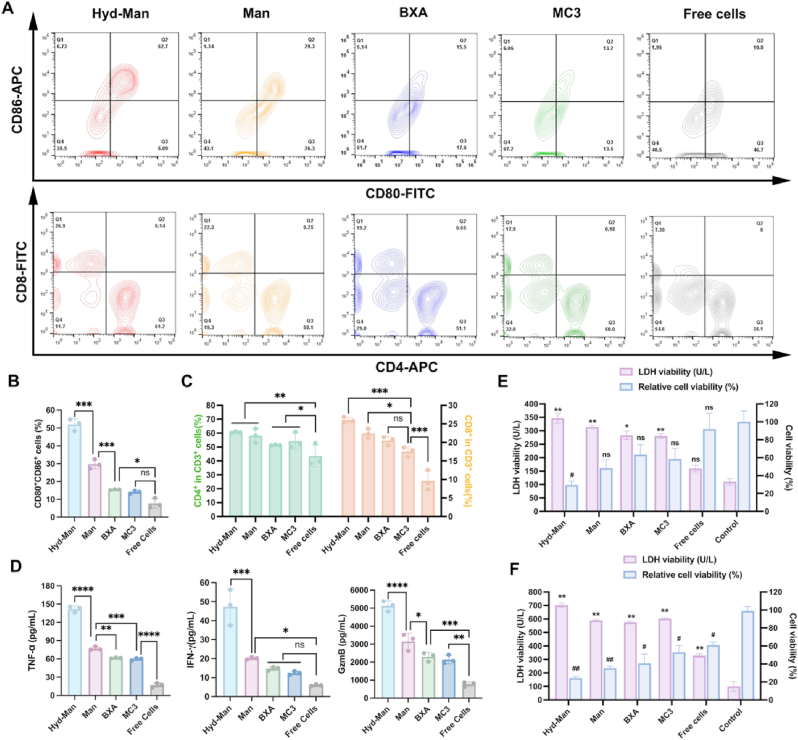
Immune activation in vitro. Flow analysis (A), and quantify of mature DC cells (B) after treated with indicated TRP2_180-188_ mRNA-encapsulated LNPs for 24 h (n = 3). (C) Flow analysis and quantify of mature T cells after co-incubated with DCs treated with indicated TRP2_180-188_ mRNA-encapsulated LNPs for 24 h (n = 3). (D) Analysis of antitumor cytokines in vitro by ELISA (n = 3). Evaluation of cytotoxic killing ability and the level of LDH expression after treated with indicated TRP2_180-188_ mRNA-encapsulated LNPs for (E) 24 h and (F) 48 h (n = 3, ∗ and # was compared with Control). Significant differences were assessed using a one-way ANOVA with Tukey test (^#^p or ∗p < 0.05; ^##^p or ∗∗p < 0.01; ^###^p or ∗∗∗p < 0.001; ^####^p or ∗∗∗∗p < 0.0001). Data were presented as mean ± SD.

To evaluate the immune activation potential of Hyd-Man LNPs, we utilized TRP2_180-188_ mRNA as a model antigen and assessed DC and T cell activation via flow cytometry. Notably, the Hyd-Man LNPs group significantly increased the count of mature DCs, exhibiting a five-fold rise compared to the MC3 LNPs group (Fig. 5A and B). The maturation rate of DCs in the Hyd-Man group was markedly higher than in the Man LNPs group (51.90 ± 3.37 % vs. 29.70 ± 2.26 %), reflecting superior mRNA delivery performance. Furthermore, mannose targeting in Man LNPs yielded a two-fold increase in mature DC cells relative to BXA LNPs.

We further examined the stimulation of CD4^+^ T helper and CD8^+^ T effector cells (cytotoxic T cells) post-treatment with various LNP groups. As illustrated in Fig. 5A and C, there was a marked increase in CD8^+^ T cells following Hyd-Man LNP treatment, with CD8^+^ T effector levels rising by 1.5-fold compared to MC3 LNPs and 2.7-fold over control groups. While in CD4^+^ T cells, it was no significant different between each LNP group. It seemed that the Hyd-Man LNP treatment was more likely to activate CD8^+^ T cells, and such phenomenon was helpful for the design of tumor vaccines. The CD8^+^ T cells directly kill tumor cells by recognizing the MHC I class molecules - antigen peptide complexes on the surface of tumor cells, and play a crucial role in immune memory, their activation was one of the core goals in the design of tumor vaccines [55]. Besides, multiple clinical studies have shown that the efficacy of tumor vaccines was closely related to the CD8^+^ T cell response [56,57].

To verify the anti-tumor immune effect of LNP, we quantified cytokine levels (IL-12, TNF-α, IFN-γ, and Granzyme B) in the culture supernatants post mRNA LNP exposure. Treatment with Hyd-Man LNPs elicited robust antitumor immune responses, as evidenced by significantly elevated levels of IL-12 (1.8−2.0-fold) (Fig. S9), TNF-α (2.3−2.4-fold), IFN-γ (3.2−3.8-fold), and Granzyme B (2.2−2.4-fold) compared to BXA and MC3 groups (Fig. 5D). Notably, the Hyd-Man group demonstrated even greater cytokine expression than the Man group, further validating the effectiveness of our pH-mediated endosomal escape strategy.

Subsequently, we evaluated the indirect antitumor efficacy of Hyd-Man LNPs through immune response activation via CCK-8 and LDH assays, which assess cell viability and LDH release, respectively. Following a 24-h incubation of BMDCs, combined with splenic lymphocytes (SLCs) and B16-F10 cells (1:5:1), Hyd-Man LNPs significantly activated antitumor immunity, resulting in the lowest cell viability and highest LDH levels among all treatment groups within 24 h or 48 h (Fig. 5E and F).

### 3.6 In vivo targeted-uptake and expression of mRNA-LNP and TRP2_180-188_-specific CTL induction

The in vivo biodistribution after each treatment was showed in Fig. S10. The major organs (heart, liver, spleen, lung, kidney, and lymph nodes) of mice were harvested for fluorescence imaging at 12 h using an In Vivo Imaging System (IVIS). Hyd-Man LNP predominantly accumulated in the LNs, followed by in the spleen, and negligible deposition was observed in the other harvested organs (i.e., heart, liver, lung, and kidney). Besides, the uptake of Man LNP in the spleen was weaker than that of Hyd-Man LNP, which was similar to the results of in vitro uptake studies (Fig. 2A). This might be due to the smaller particle size of Hyd-Man.

**Fig. 6:**
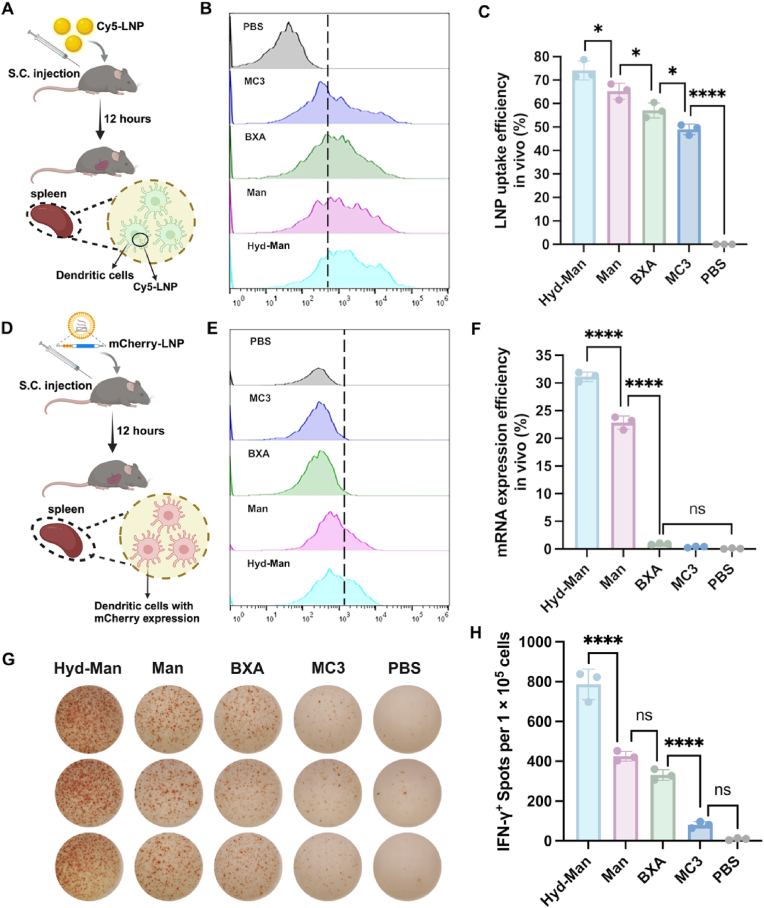
In vivo uptake and expression of mRNA and CTL induction. (A) Schematic diagram of cellular uptake of Cy5-labeled LNPs in vivo. Created with BioRender.com. (B) In vivo uptake analysis by flow cytometry analysis and (C) quantify of Cy5-LNP events in splenic DCs (n = 3). (D) Schematic diagram of mRNA expression efficiency of mCherry mRNA encapsulated in different LNPs in vivo. Created with BioRender.com. (E) In vivo expression efficiency analysis by flow cytometry analysis and (F) quantify of mCherry fluorescence events in DCs (n = 3). (G) ELISpot images and (H) spot nos. of IFN-γ-secreting T cells within SLCs of vaccinated mice (n = 3). Significant differences were assessed using a one-way ANOVA with Tukey test (∗p < 0.05; ∗∗p < 0.01; ∗∗∗p < 0.001; ∗∗∗∗p < 0.0001). Data were presented as mean ± SD.

To further analyze the DC targeting ability in vivo, we assessed the LNP uptake and mRNA expression efficiency of Hyd-Man LNP in DCs derived from spleen at 12 h post-subcutaneous injection (Fig. 6A and D). As illustrated in Fig. 6B and C, there was an improved cellular uptake efficiency in DCs following Hyd-Man and Man LNPs treatment, with Cy5-labeled LNP events rising by 1.3-fold and 1.1-fold compared to BXA LNPs, respectively. Interestingly, the Hyd-Man LNP group demonstrated significantly enhanced in vivo uptake behavior, consistent with the in vitro findings (Fig. 2A). It seems that the addition of Hyd-mPEG_2000_ resulted in smaller LNP particle size, which may facilitate more efficient cellular uptake. Additionally, the lymph nodes were also studied using the same procedure, and Hyd-Man LNP group also exhibited similar results to those of the DCs uptake in spleen (Fig. S11).

Besides, the co-localization of Cy5-LNP and CD11c^+^ DC cells in lymph nodes was analyzed using cryostat section combined with tissue immunofluorescence staining, and the co-localization coefficient (co-localization ratio) of Cy5-LNP and CD11c^+^ DC was evaluated. Here, the co-localization ratio was closer to 1, indicating a higher degree of co-localization. As shown in Fig. S12, the co-localization ratio of CD11c^+^ DC with Cy5-LNP in each group were as follows: 0.80 ± 0.10 (Hyd-Man), 0.75 ± 0.08 (Man), 0.61 ± 0.02 (BXA), 0.56 ± 0.07 (MC3). This results further demonstrated that due to the targeting effect of mannose, Hyd-Man LNP and Man LNP were more efficiently taken up by DCs in the lymph nodes after subcutaneous injection.

To investigate the in vivo mRNA expression efficiency facilitated by LNPs in each group, we quantified mCherry fluorescence events in splenic DCs. The results indicated most stronger expression in the Hyd-Man group, with faint effects from BXA and MC3 treatment (Fig. 6E and F). Furthermore, Hyd-Man LNP treatment exhibited significant mRNA expression efficiency than Man LNP, indicating successful pH-mediated release of mRNA from endosomes in vivo.

Further, the levels of IFN-γ-secreting T cells within SLCs were evaluated by an ELISpot assay in Fig. 6G and H to assess the TRP2_180-188_ peptide (SVYDFFVWL)-specific CTL response in vivo. All the vaccinated groups generated IFN-γ-secreting T cells to some extent, indicating the robust T cells response generated by the mRNA vaccine. Notably, treatment with Hyd-Man LNPs elicited robust antitumor immune responses, as evidenced by significantly elevated spots of IFN-γ compared to other groups (Fig. 6H). Similar to the percentage of SVYDFFVWL-specific CD8^+^ T cells, the mice without vaccination showed feeble response to the stimulation of SVYDFFVWL.

### 3.7 Tumor immune response and Inhibition of melanoma growth via Hyd-Man LNPs/TRP2_180-188_ with αPD-L1

**Fig. 7:**
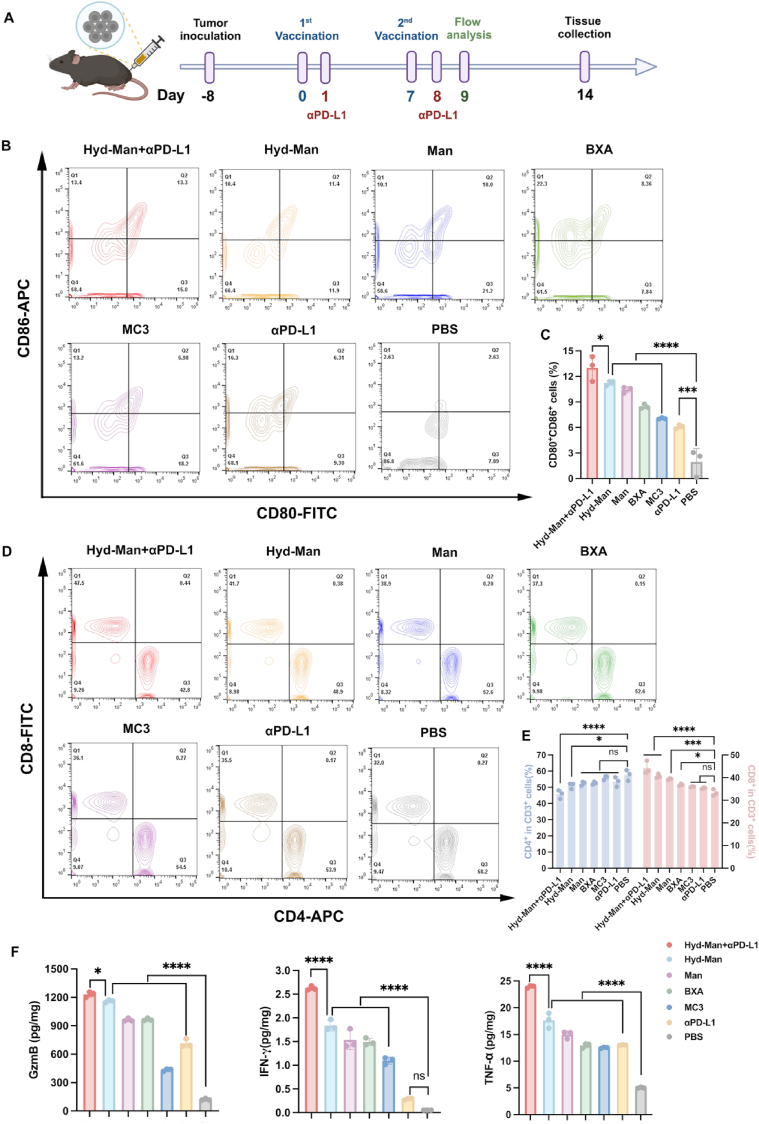
In vivo antitumor immune responses by indicated treatments in the B16-F10 melanoma model. (A) Schematic representation of the administration strategy in the B16-F10 mouse model. Created with BioRender.com. (B) Flow cytometry analysis and (C) quantify the percentage of DC cells (CD80^+^CD86^+^). (D) Flow cytometry analysis and (E) quantify the percentage of T cells (CD3^+^CD4^+^, CD3^+^CD8^+^) (n = 3). (F) ELISA analysis of TNF-α, IFN-γ, and GzmB expression in 200 mg of B16-F10 tumor tissues (n = 3). Significant differences were assessed using a one-way ANOVA with Tukey test (∗p < 0.05; ∗∗p < 0.01; ∗∗∗p < 0.001; ∗∗∗∗p < 0.0001). Data were presented as mean ± SD.

To further confirm the therapeutic efficacy of Hyd-Man LNPs and the synergistic effect of αPD-L1, intervention treatment of Hyd-Man LNPs/TRP2_180-188_ in an established B16-F10 tumor model was assessed. Following the administration of 1 × 10^5^ B16-F10 cells subcutaneously on day −8, vaccination was conducted as outlined in Fig. 7A, complemented by αPD-L1 injection on days 1 and 8 to mitigate immunosuppression. Flow cytometric analyses performed on day 9 indicated that the Hyd-Man+αPD-L1 group exhibited a superior activation of DCs within tumors compared to Hyd-Man alone (Fig. 7B and C). While most vaccinated mice demonstrated increased CD8^+^ T cells in tumors versus PBS controls, the combination of Hyd-Man with αPD-L1 yielded comparably elevated responses (Fig. 7D and E).

By day 14, cytokine levels (TNF-α, IFN-γ, Granzyme B) within tumor tissues were analyzed using ELISA (Fig. 7F). All vaccinated groups showed enhanced cytokine expressions, with the Hyd-Man+αPD-L1 group achieving remarkable outcomes compared to others. Notably, combining αPD-L1 treatment resulted in substantial enhancements in cytokine levels, affirming its synergistic effects.

**Fig. 8:**
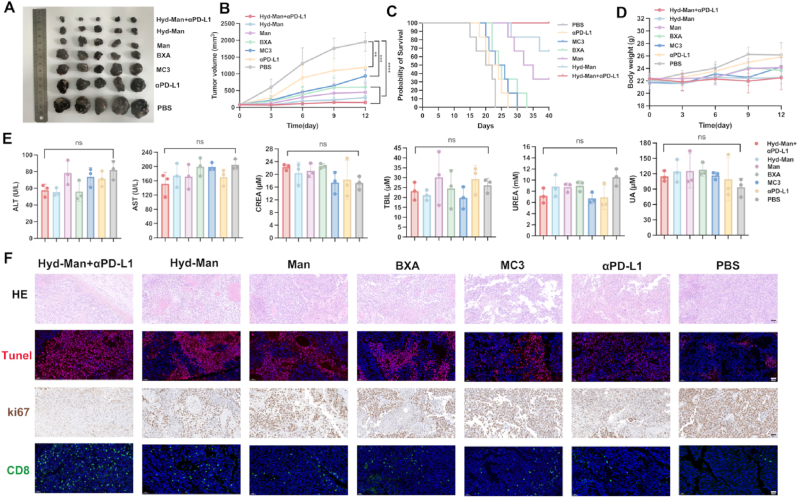
Antitumor activity of indicated treatments in the B16-F10 melanoma model. (A) Representative images and (B) volumes of B16-F10 tumors (n = 5). (C) Survival curves of B16-F10 tumor-bearing mice treated with various therapeutic combinations (n = 6). In vivo safety assessment following administration of the indicated treatment groups compared with PBS group through (D) changes in body weight (n = 5), and (E) serum biochemical analysis of ALT, AST, CREA, TBIL, UREA and UA (n = 3). Histopathological analysis in tumor tissues from each experimental cohort utilizing (F) H&E, TUNEL, ki67, and CD8 staining methodologies. Scale bar = 50 μm. Significant differences were assessed using a one-way ANOVA with Tukey test (∗p < 0.05; ∗∗p < 0.01; ∗∗∗p < 0.001; ∗∗∗∗p < 0.0001). Data were presented as mean ± SD.

Post-vaccination analysis revealed that Hyd-Man+αPD-L1 treatment dramatically reduced tumor volume and improved survival rates relative to other treatment groups (Fig. 8A–C). The tumor growth curves indicated rapid progression in the PBS group, with marginal effects from αPD-L1 treatment alone. However, Hyd-Man treatment effectively controlled tumor growth, with a marked increase in efficacy observed when coupled with αPD-L1. The Hyd-Man+αPD-L1 group displayed a 100 % survival rate in B16-F10-bearing mice after 40 days (Fig. 8C). Furthermore, body weight across all groups remained stable, suggesting favorable safety profiles (Fig. 8D).

Histopathological evaluations (H&E staining) of major organs indicated no significant differences in histology or serum biochemical parameters compared to PBS, confirming the safety of our therapeutic approach (Fig. 8E–S13). Histological analysis of tumor sections using H&E, Ki67, and TUNEL staining demonstrated substantial nuclear fragmentation and apoptosis within both the Hyd-Man and Hyd-Man+αPD-L1 treatment groups, with greater effects observed in the latter (Fig. 8F). Enhanced CD8^+^ T cell activation within tumor tissues was likewise evident in the Hyd-Man+αPD-L1 cohort, indicating a considerable immune response (Fig. 8F). These findings provide compelling evidence for the potent antitumor immune responses elicited by TRP2_180-188_ mRNA-encapsulated Hyd-Man LNPs, particularly when combined with αPD-L1 therapy through an integrated strategy of DC-targeting and pH-mediated endosomal escape.

## 4 Conclusion

In summary, we successfully developed a mannose-modified and pH-responsive mRNA vaccine, achieving targeted delivery to DCs and facilitating efficient endosomal escape—an approach that significantly enhances antitumor immunity. The Hyd-Man mRNA vaccine was rapidly taken up by DCs, ensuring efficient release into the cytoplasm for robust mRNA expression within 4 h. Through assessments of escape efficiency and relevant protein expression profiles, we demonstrated the effectiveness of the cleavable PEG-lipid (Hyd-mPEG_2000_), which directly promoted early endosomal escape and immune response activation. Notably, when compared to commercially available mRNA vaccines (e.g., MC3), Hyd-Man mRNA vaccines exhibited superior therapeutic efficacy, particularly in conjunction with αPD-L1.

## CRediT authorship contribution statement

**Sizhen Wang:** Writing – original draft, Methodology, Investigation. **Jianyu Zheng:** Methodology, Investigation. **Jiao Zhou:** Methodology, Investigation. **Weiwei Jiang:** Visualization. **Zhendong Chen:** Visualization. **Xiaoxian Wu:** Visualization. **Beibei Guo:** Writing – review & editing, Funding acquisition. **Yanfeng Wu:** Writing – review & editing. **Feng Yang:** Writing – review & editing, Funding acquisition, Conceptualization.

## Declaration of competing interest

The authors declare that they have no known competing financial interests or personal relationships that could have appeared to influence the work reported in this paper.

## Data Availability

Data will be made available on request.
