# Supplementary material for: Engineered lipid nanoparticles with synergistic dendritic cell targeting and enhanced endosomal escape for boosted mRNA cancer vaccines
Source: Mater Today Bio. 2025 Jul 19;34:102107. doi: 10.1016/j.mtbio.2025.102107 (PMC12308020; doi:10.1016/j.mtbio.2025.102107)
Supplement: Multimedia component 1 [file mmc1.docx]

Supplementary Materials for

**Engineered Lipid Nanoparticles with Synergistic Dendritic Cell Targeting and Enhanced Endosomal Escape for Boosted mRNA Cancer Vaccines**

Sizhen Wang^1, *^, Jianyu Zheng^1, *^, Jiao Zhou^1, *^, Weiwei Jiang^1^, Zhendong Chen^1^, Xiaoxian Wu^1^, Beibei Guo^1, #^, Yanfeng Wu^2, #^, Feng Yang^1,2, #^

^1^ National Key Laboratory of Immunity and Inflammation & School of Pharmacy, Naval Medical University, Shanghai, 200433, China.

^2^ National Key Laboratory of Immunity and Inflammation & Institute of Immunology, College of Basic Medical Sciences, Naval Medical University, Shanghai, 200433, China.

^#^ Corresponding author. Email: yangfeng1008@126.com (F.Y.), wuyf@immunol.org (Y.W.), bbguo1994@163.com (B.G.).

^*^ These authors contributed equally.

1. **Supplementary Text**

**1.1 Synthesis of benzene-1,3,5-tricarboxamide ionizable lipids (BXA)**

The BXA ionizable lipid was prepared according to previous study [1] and the synthetic route was described in **Figure.S1**. Briefly, 1,3,5-benzotricarboxylic chloride (1.88 mmol) was dissolved in dichloromethane (CH_2_Cl_2_), and 10 mL of pyridine was added under ice bath conditions and stirred. Boc-1,3-propanediamine in 30 mL of dichloromethane was then added drop-wise to the above system with stirring. After completion of the addition, the mixture was allowed to stir at room temperature for 24 h. The product was purified by column chromatography using a dichloromethane/methanol solvent system to yield **Compound 1**.

Compound 1 was dissolved in CH_2_Cl_2_, and trifluoroacetic acid (TFA) was added under ice bath conditions followed by stirring at room temperature until the reaction completion. The resulting mixture underwent distillation, followed by dissolution in methanol and concentration through distillation. **Compound 2** was obtained after recrystallization from ethyl acetate (EtOAc) and subsequent concentration via distillation.

Compound 2 (1 mmol) in tetrahydrofuran (THF) was stirred with triethylamine for one hour before adding N-decanal (9 mmol) and triacetoxyborohydride (NaBH[OAc]_3_, 5 mmol) under nitrogen protection for stirring at room temperature over 72 h. Purification via column chromatography utilizing a CH_2_Cl_2_/MeOH/triethylamine solvent system yielded the target compound **BXA**. The product BXA ionizable lipid was characterized and verified by mass spectrum (MS) and nuclear magnetic resonance spectrum (^1^H NMR) **(Figure.S3)**. The actual mass-charge ratio (m/z) of the prepared BXA ionizable lipid is 1178.1371**(Figure.S3A)**, which is the same as the theoretical one (1178.13). Additionally, each chemical group had its own corresponding chemical shift (ppm) in ^1^H NMR analysis **(Figure.S3B)**, which further verified the successful synthesis of BXA.

**1.2 Synthesis of pH-responsive hydrazone-based mPEG_2000_ lipid (Hyd-mPEG_2000_)**

The synthetic route was described in **Figure.S2**. Briefly, adiponitrile dihydrazide (5.7 mmol) was dissolved in deionized water while Di-tert-butyl dicarbonate (5.7 mmol) was dissolved in methanol. Blending and adjusting the pH of the mixture to 8-9 and keep stirring overnight at room temperature. Extracted with ethyl acetate, and collected the aqueous phase and evaporated, then adjust its pH to neutrality and extracted with ethyl acetate again. Collected and evaporated the organic phase to obtain a colorless and transparent liquid **(Compound 1)**. Compound 1 was characterized and verified by nuclear magnetic resonance spectrum (^1^H NMR) **(Figure.S4)**.

Cholesteryl succinate monoester (0.41 mmol) was dissolved in CH_2_Cl_2_ and added with a mixture of EDCI (0.492 mmol), HOBT (0.492 mmol), and DIPEA (0.574 mmol) in an ice bath, keeping stirring for 30 min. NH_2_-PEG_400_-COOH (0.21 mmol) was then introduced, and the reaction proceeded for 24 h. The product was washed three times before extraction of the organic phase using CH_2_Cl_2_. The residue was purified by column chromatography with gradient elution from 100% CH_2_Cl_2_ to CH_2_Cl_2_/MeOH/ formic acid (20/1/0.1 by volume) to give **Compound 2**.

Compound 2 (0.0786 mmol) was dissolved in CH_2_Cl_2_, and then EDCI (0.0943 mmol), HOBT (0.0943 mmol), and DIPEA (0.1179 mmol) were added to the mixture in an ice bath and keep stirring for 1 h. Then, Compound 1 (0.1179 mmol) was added, and the reaction was allowed to proceed for 24 h. The product was then washed and CH_2_Cl_2_ was used to extract the organic phase. After the solvent was removed, the residue was purified by column chromatography with gradient elution from 100% CH_2_Cl_2_ to CH_2_Cl_2_/MeOH (20/1 by volume) to obtain **Compound 3**.

Compound 3 (0.48 mmol) was dissolved in CH_2_Cl_2_, and TFA was added in an ice bath. The mixture was keep stirring for 17 h at room temperature and neutralized with saturated NaHCO_3_ in an ice bath, diluted and extracted with CH_2_Cl_2_. The organic phase was collected and evaporated to obtain **Compound 4**, then reacted with benzaldehyde-mPEG_2000_ (mPEG_2000_-DF, R-BJQPE-2k, Xi’an ruixi Biological Technology Co., Ltd, China) in DMF for 24 h. After that, the mixture was dialyzed in an ice bath for 24-48 h to give the target compound **Hyd-mPEG_2000_**, and it was characterized and verified by nuclear magnetic resonance spectrum (^1^H NMR) **(Figure.S5)**.

**1.3 Measurement of Hyd-PEG_2000_ Modification Efficiency**

Hyd-PEG_2000_ modification efficiency was quantified by detecting remaining primary amine groups using TNBS [2]. The Hyd LNP solution was diluted with 0.1 M borate buffer (pH 9.5) and treated with 1% TNBS for 60 min. Following treatment, 10% Triton X-100 was added to a final concentration of 1.0% (w/v), and absorbance was measured at 420 nm, normalizing against Compound 4. Modification efficiency was calculated as follows:

Modification Efficiency (ME%) = 1 - (A_U_-A_U0_) / (A_T_-A_T0_) × 100%

A_U_ is the absorbance of the Hyd LNP solution, A_U0_ is the blank solvent absorbance, A_T_ is the absorbance of Compound 4, and A_T0_ is the corresponding blank solvent absorbance.

**1.4 Isolation of Mouse BMDCs and SLCs**

Six-week-old C57BL/6 mice were euthanized and processed using 75% ethanol to sterilize femurs and tibias. Bone marrow (BM) cells were isolated by flushing the bones with RPMI-1640. To differentiate into bone marrow-derived dendritic cells (BMDCs), BM cells were cultured in RPMI-1640 supplemented with 10% FBS, 100 U/ml penicillin-streptomycin, 20 ng/ml murine granulocyte-macrophage colony-stimulating factor (GM-CSF, MedChemExpress, USA), and 10 ng/ml IL-4 (MedChemExpress, USA). Non-adherent cells were collected on day 3 and used for experiments starting at day 6. Spleen lymphocytes (SLCs) were isolated from the spleen of tumor-bearing mice using a 100 µm cell strainer followed by Ficoll separation.

**1.5 In vivo Biodistribution with IVIS**

The in vivo biodistribution after LNP treatment was researched using the In Vivo Imaging System (IVIS). In this research, 1,1'-Dioctyl-3,3,3',3'-tetramethylindole iodide (DiR) was used to label Hyd-Man LNP, at 1% of the total lipid volume. The other LNP groups were labeled with the same amount of DiR as the Hyd-Man LNP group. Male C57BL/6 mice (20-22 g) were treated subcutaneously with PBS or DiR-labeled LNPs (10 μg/mL DiR). Main organs (heart, liver, spleen, lung, kidney, and lymph nodes) were collected for analysis at 12 hours post-subcutaneous injection.

**1.6 Targeted-Uptake In Vivo**

Male C57BL/6 mice (20-22 g) were treated with PBS or Cy5-labeled LNPs (200 ng/mL Cy5). Spleen and lymph nodes were collected and cells extracted for flow cytometric analysis of the events of Cy5-labeled LNP uptake by DCs (CD11c^+^) at 12 hours post-subcutaneous injection. The co-localization of DC cells (CD11c^+^, green) and Cy5-LNP (Cy5, yellow) in lymph nodes of each group was analyzed using a combined technique of frozen sectioning and immunofluorescence staining.

**1.7 mRNA Expression In Vivo**

Male C57BL/6 mice (20-22 g) were treated with PBS or mCherry-encapsulated LNPs (600 ng mRNA/mL). Spleen was collected and SLCs extracted for flow cytometric analysis of the number of DCs (CD11c^+^) that expressing mCherry protein at 12 hours post-subcutaneous injection.

**1.8 ELISpot Assay**

Before vaccination, male C57BL/6 mice (20-22 g) were subcutaneously implanted with 1 × 10^5^ B16-F10 cells, and treated with PBS or TRP2_180-188_ mRNA-encapsulated LNPs (5 μg mRNA/per mouse) on day 5 and 12. One week after the second vaccination, SLCs were extracted and suspended in RPMI-1640 medium containing 10% fetal bovine serum. The ELISpot assay was conducted using the Mouse Interferon-γ ELISPOT Kit (DAKEWE, #2210005, China). Then 1 × 10^5^ SLCs cells were incubated in RPMI-1640 medium with 5 μg/mL of TRP2_180-188_ peptide (SVYDFFVWL) at 37 °C for 20 h and followed by procedure. The pictures were then taken, and spot nos. of each sample were calculated automatically.


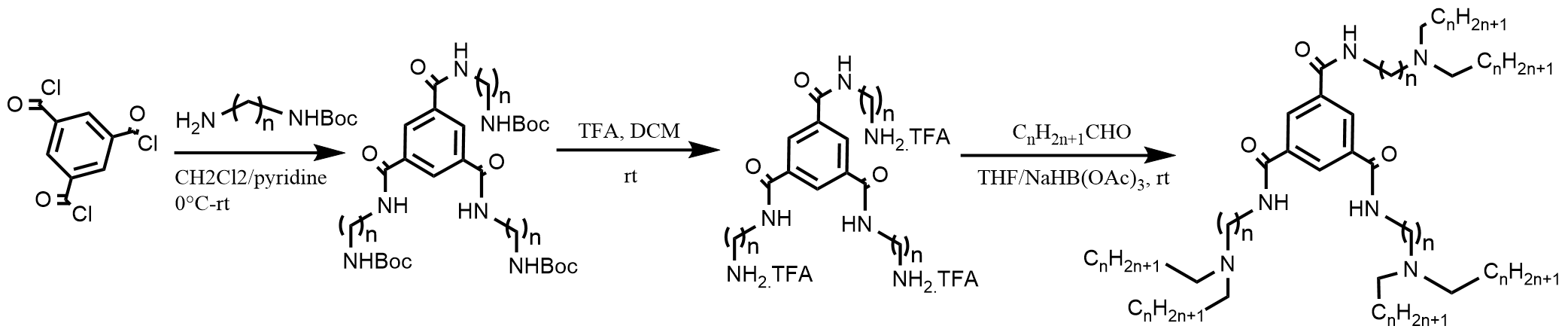


Figure S1. The synthetic route of BXA ionizable lipids.


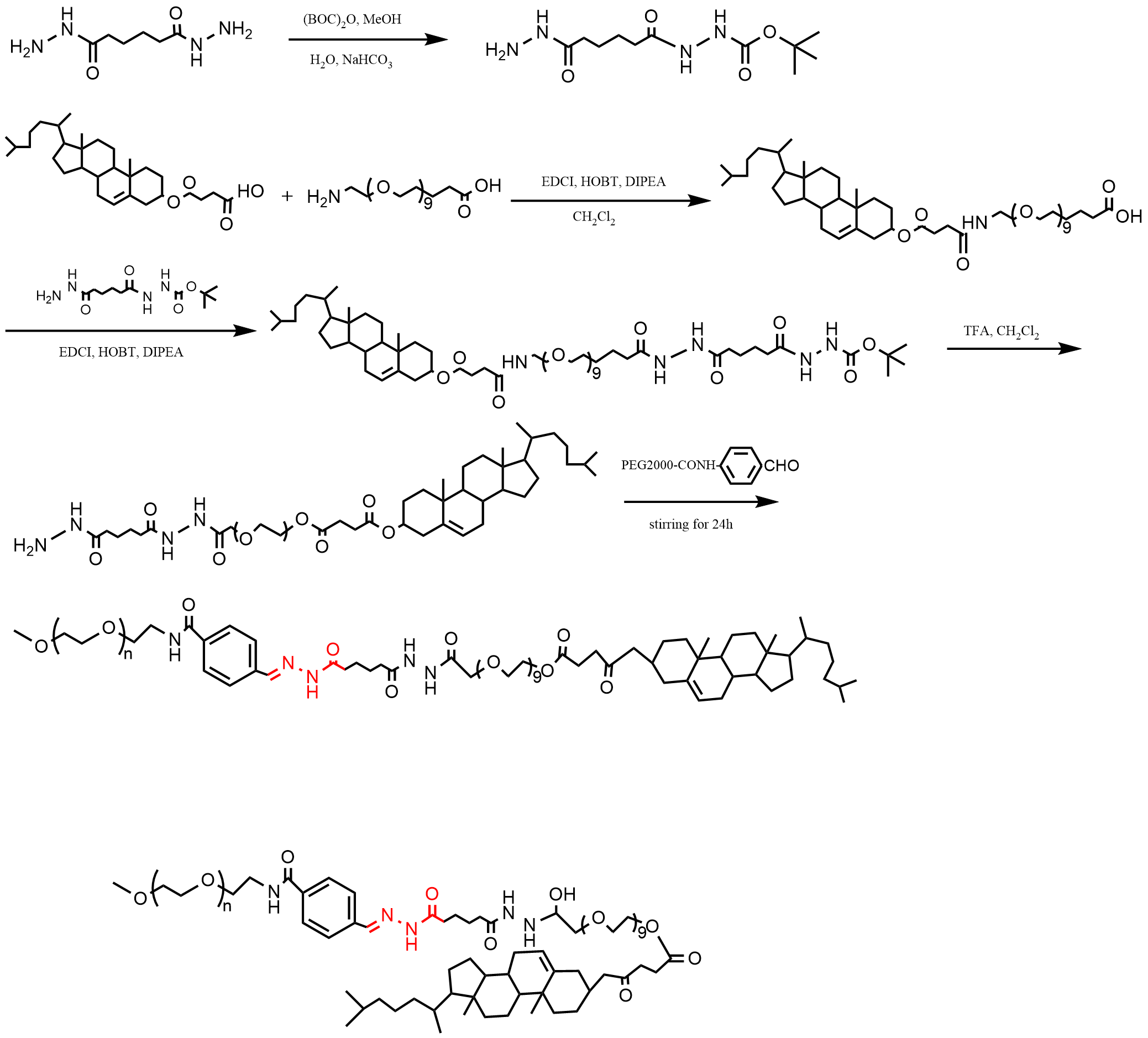


Figure S2. The synthetic route of Hyd-mPEG_2000_.


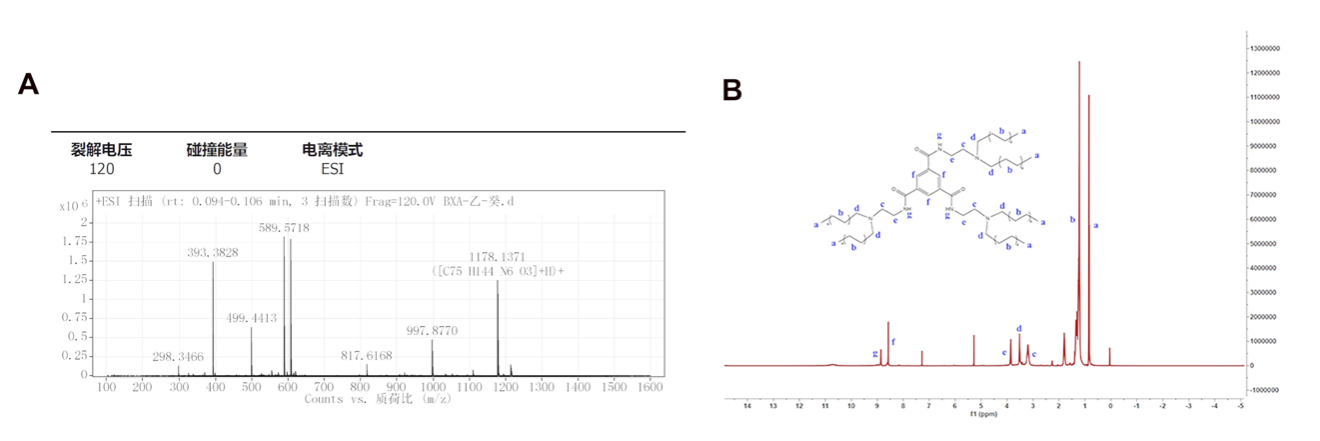


Figure S3. Molecular structure identification of BXA ionizable lipid by (A) MS and (B) ^1^H NMR.


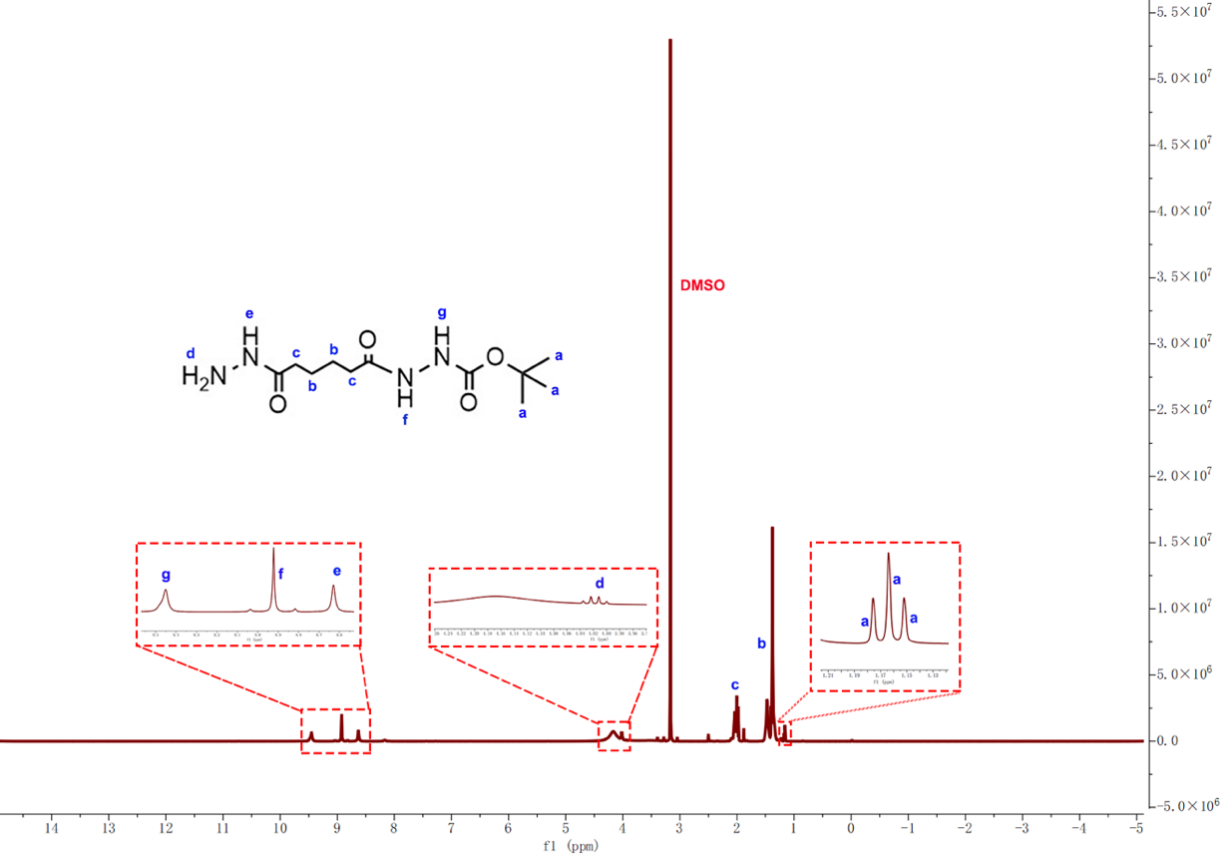


**Figure S4.** Molecular structure identification of Compound 1 by ^1^H NMR.


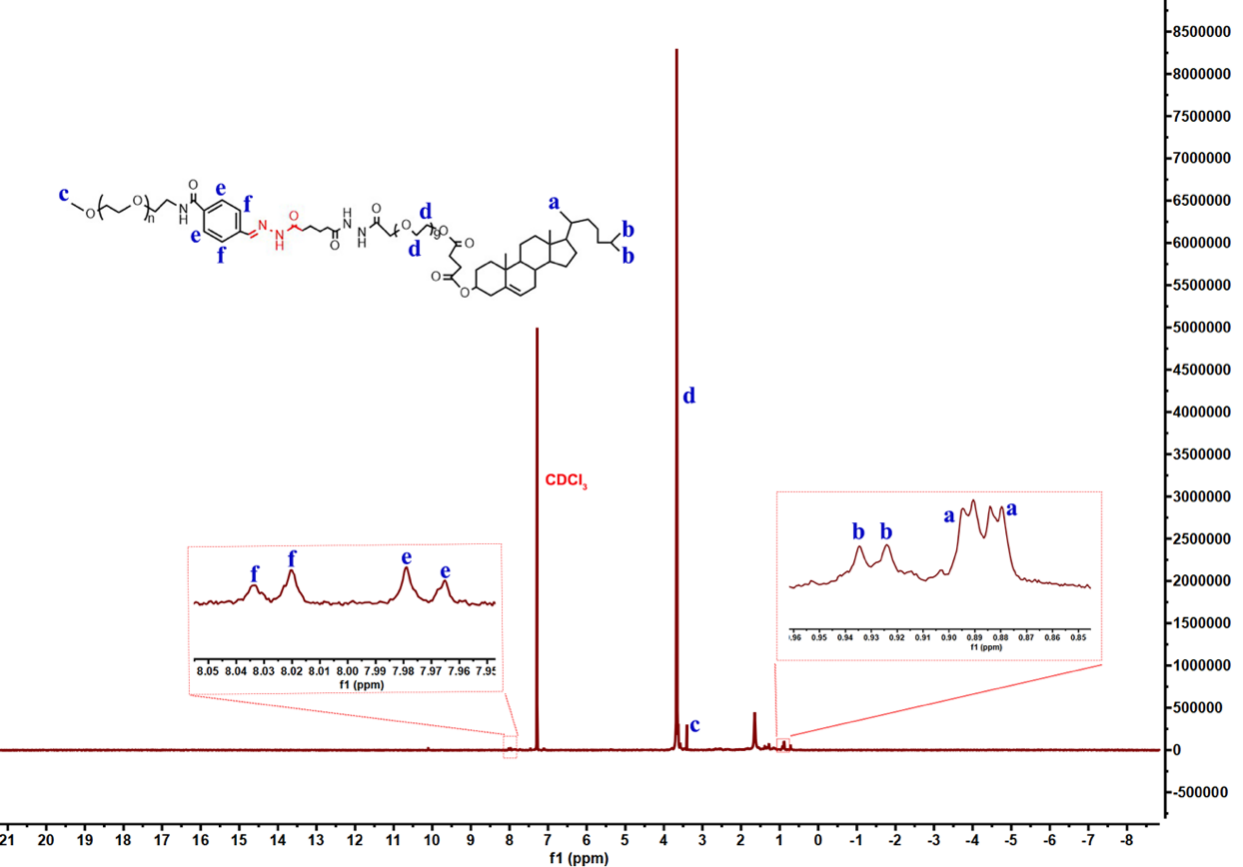


**Figure S5.** Molecular structure identification of Hyd-mPEG_2000_ by ^1^H NMR.


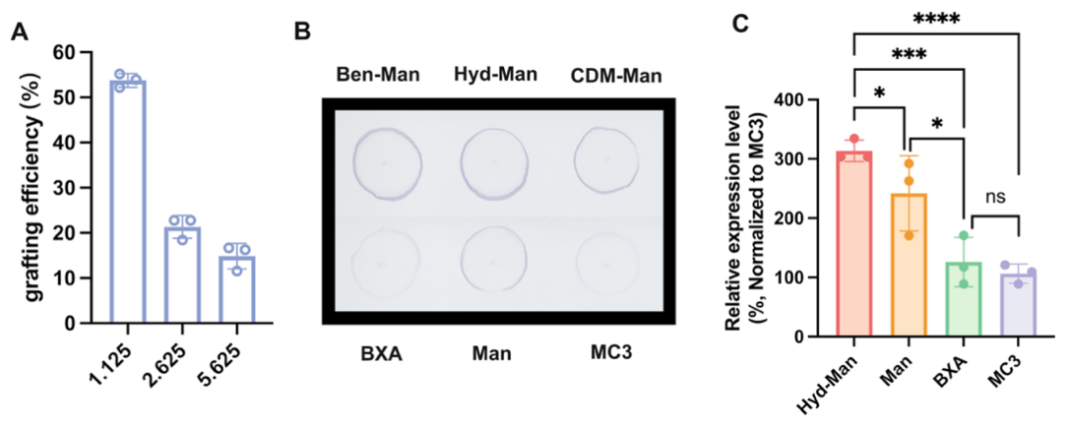


**Figure S6.** (A) Grafting efficiency of Hyd-mPEG_2000_ with different molar ratio. (B) Transfection efficiency analysis of different TRP2_180-188_ mRNA-encapsulated LNPs by Dot blot. (C) Quantitative analysis of TRP2_180-188_ mRNA-encapsulated LNPs transfection efficiency by Dot blot (n = 3). Significant differences were assessed using a one-way ANOVA with Tukey test (^*^p < 0.05; ^**^p < 0.01; ^***^p < 0.001; ^****^p < 0.0001). Data were presented as mean ± SD.


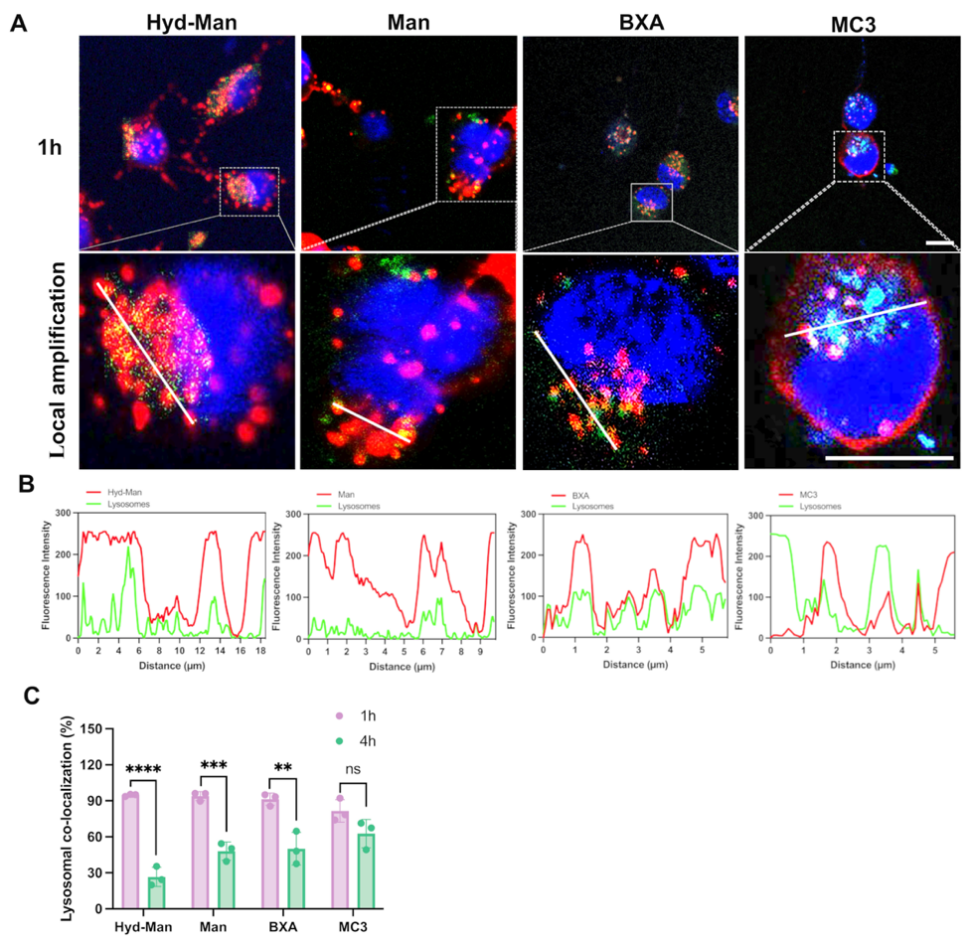


**Figure S7.** (A) Colocalization of Cy5-labeled mCherry mRNA-encapsulated LNPs (red) with lysosome (green) at 1 h. Scale bar = 10 µm. (B) Plot profile analysis of local amplification with the mean fluorescence intensity LNPs (red) and lysosomes (green) in the underlined area at 1 h by Image J software. (C) Quantitative analysis of colocalization between LNPs and lysosomes at 1 h and 4 h (n = 3). Significant differences were assessed using a one-way ANOVA with Tukey test (^*^p < 0.05; ^**^p < 0.01; ^***^p < 0.001; ^****^p < 0.0001). Data were presented as mean ± SD.


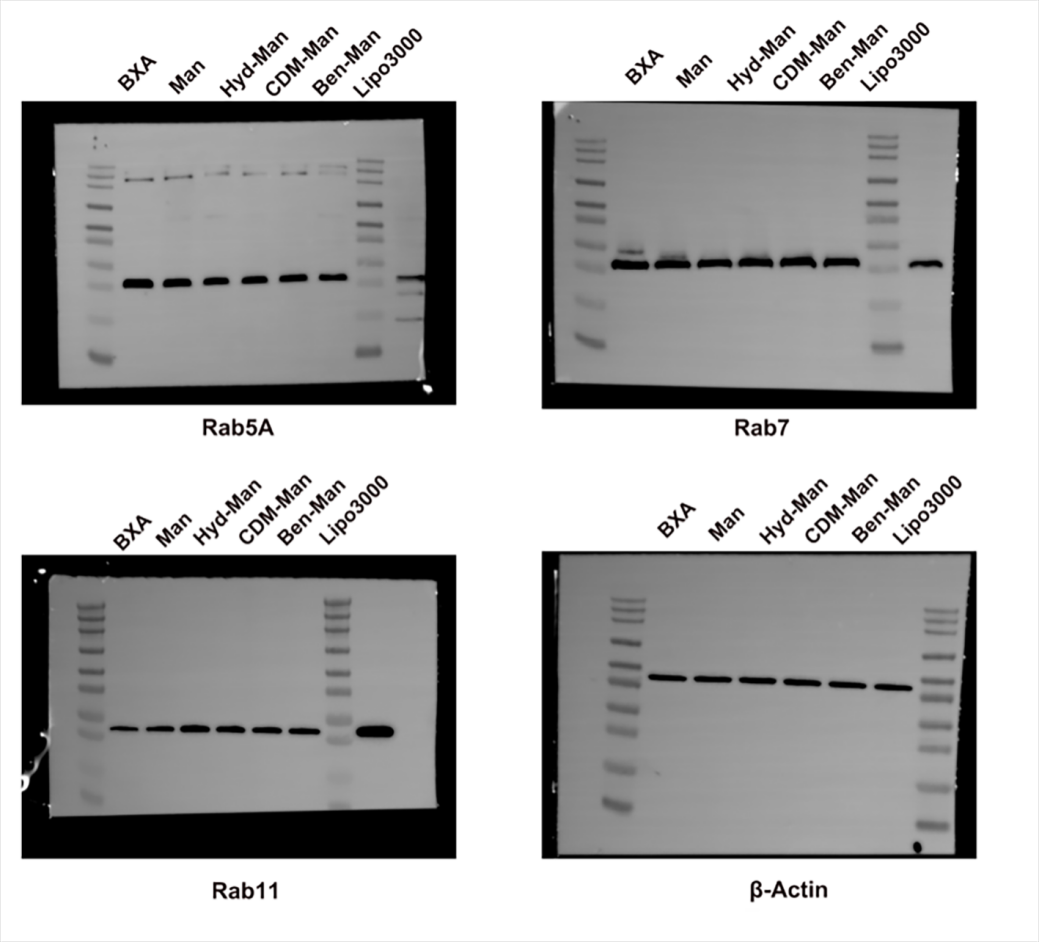


**Figure S8.** Uncropped gels for Western Blot.


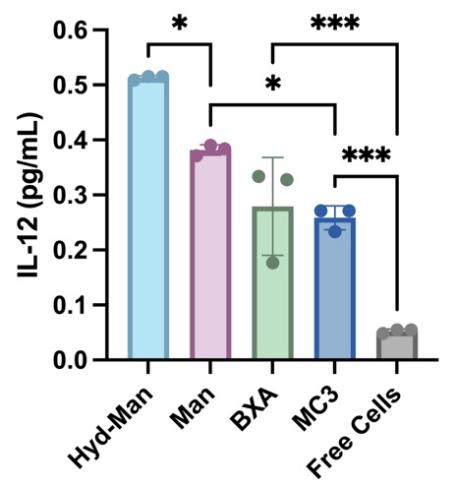


**Figure S9.** Analysis of antitumor cytokines in vitro by IL-12 ELISA (n = 3). Significant differences were assessed using a one-way ANOVA with Tukey test (^*^p < 0.05; ^**^p < 0.01; ^***^p < 0.001; ^****^p < 0.0001). Data were presented as mean ± SD.


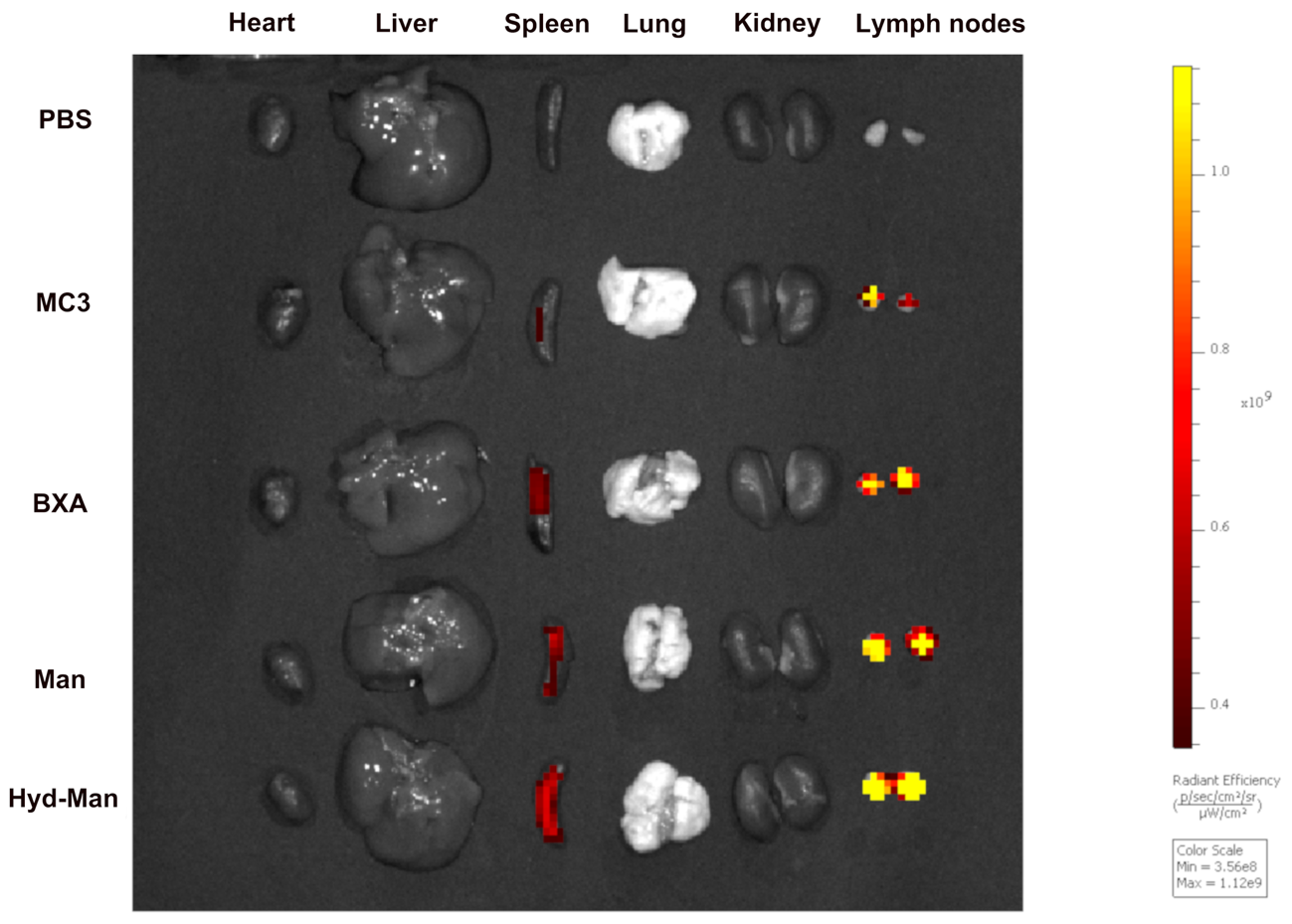


**Figure S10.** The in vivo biodistribution of each group with IVIS.


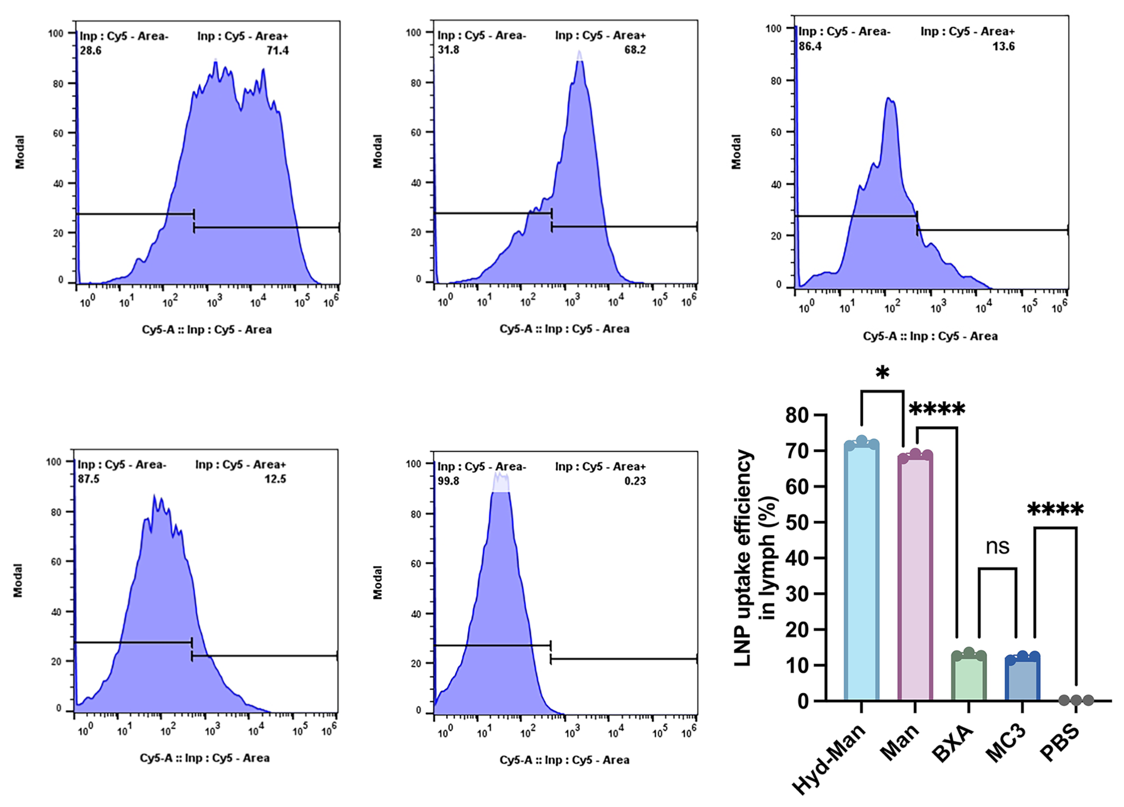


**Figure S11.** In vivo uptake analysis by flow cytometry analysis and quantify of Cy5-LNP events in DCs from the lymph nodes (n = 3). Significant differences were assessed using a one-way ANOVA with Tukey test (^*^p < 0.05; ^**^p < 0.01; ^***^p < 0.001; ^****^p < 0.0001). Data were presented as mean ± SD.


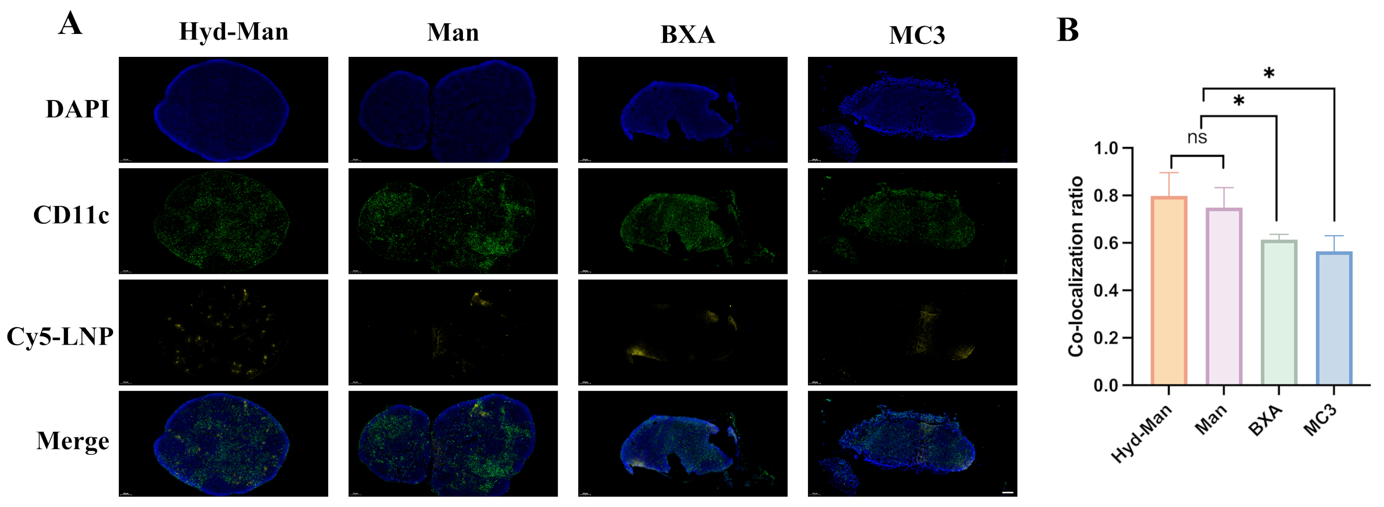


**Figure S12.** (A) The co-localization of Cy5-LNP with DC cells in lymph nodes was detected by immunofluorescence technique, and (B) the co-localization was analyzed for (A) (Scale = 200 μm, n = 3, x ± SD, p^*^ < 0.05).


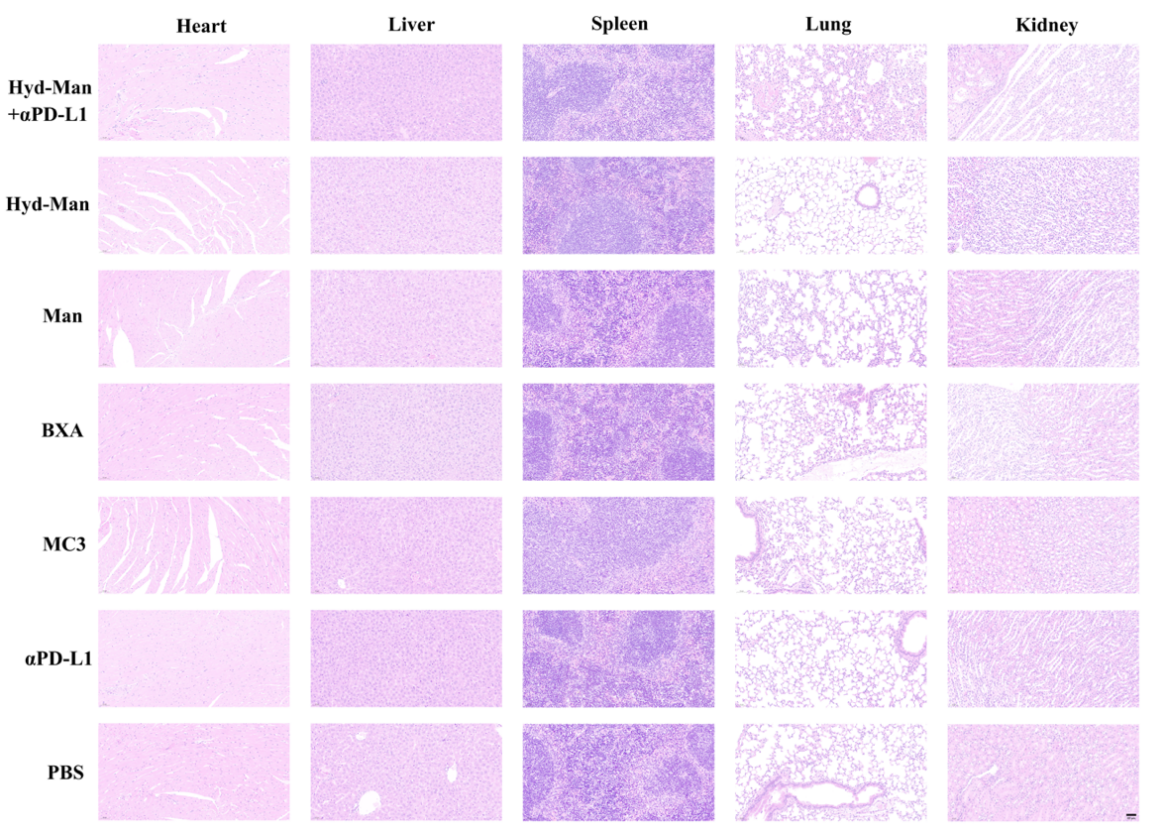


**Figure S13.** H&E staining analysis of important tissues (Scale bar = 50 µm).

Table S1. The physicochemical properties of LNPs

| Formulation | Size (nm) | PDI | Zeta potential (mV) | EE (%) |
| --- | --- | --- | --- | --- |
| BXA | 272.2 ± 3.02 | 0.11 ± 0.02 | 4.21 ± 0.71 | 96.48 ± 0.06 |
| Man | 247.0 ± 6.00 | 0.15 ± 0.02 | 4.80 ± 0.46 | 96.55 ± 0.07 |
| Hyd-Man | 123.2 ± 6.02 | 0.13 ± 0.03 | -2.95 ± 0.83 | 89.39 ± 0.21 |

Reference

[1] B. Li, X. Luo, B. Deng, J. Wang, et al. An Orthogonal Array Optimization of Lipid-like Nanoparticles for mRNA Delivery in Vivo. *Nano Lett* **2015**;15: 8099-8107.

[2] K. Hashiba., Y. Sato., H. Harashima. pH-labile PEGylation of siRNA-loaded lipid nanoparticle improves active targeting and gene silencing activity in hepatocytes. *J Control Release* **2017**; 262:239-246.
